# Association Between Observed Climate Change and Cardiovascular Disease in the United States

**DOI:** 10.1029/2025GH001588

**Published:** 2026-04-13

**Authors:** R. Yeager, C. Tuholske, M. H. E. M. Browning, C. Mattingly, S. Olmsted, A. Ossola, D. H. Locke

**Affiliations:** ^1^ Division of Environmental Medicine Department of Medicine University of Louisville Louisville KY USA; ^2^ Christina Lee Brown Envirome Institute University of Louisville Louisville KY USA; ^3^ Center for Integrative Environmental Health Sciences University of Louisville Louisville KY USA; ^4^ Department of Earth Sciences Montana State University Bozeman MT USA; ^5^ Geospatial Core Facility Montana State University Bozeman MT USA; ^6^ Department of Parks, Recreation, and Tourism Management Clemson University Clemson SC USA; ^7^ Department of Plant Sciences University of California Davis Davis CA USA; ^8^ United States Department of Agriculture Forest Service Northern Research Station Baltimore Field Station Baltimore MD USA

**Keywords:** climate change, cardiovascular, anomaly, climate change and health, CVD, stroke

## Abstract

Climate change is affecting nearly all social and environmental determinants of health. However, the extent of resulting cumulative impacts on cardiovascular and other noncommunicable diseases is not known. To address this gap and inform future research, we conducted an ecological evaluation of associations between 14 long‐term climate anomaly metrics and prevalence of coronary heart disease (CHD) and stroke in the United States. We calculated long‐term anomaly metrics as the difference between their respective 1970–1979 baseline mean and 2013–2022 modern mean values. With fitted random effects regression analysis, we evaluated associations between each anomaly metric, individually and collectively, and census tract‐level 2020–2022 mean prevalence of CHD and stroke. In adjusted models with all noncollinear anomaly metrics, we found a net association of all metrics with 0.59% higher CHD prevalence and 0.88% higher stroke prevalence (9.8% and 27.9% relative prevalence, respectively). We found the largest significant effect for location‐based annual mean air temperature anomaly, but smaller significant associations with changes in humidity, rainfall, sunlight, wind, and atmospheric pressure anomalies. We observed similar associations between mean summer and daytime heat index anomalies, which were larger than heat waves and temperature variability. While our ecological analysis cannot evaluate causal effects of climate change on cardiovascular disease, our findings align with previous research and may reflect the potential nature and extent of cumulative “slow burn” chronic health impacts of climate change among developed countries. These findings may inform precautionary policy considerations regarding the potentially large and permanent impacts of climate policies on global health.

## Plain Language Summary

Climate change affects the long‐term health of people in many ways. Much of the past research has focused on the health effects of severe heat and disasters. However, climate change has wide‐ranging impacts on health. Even after accounting for health influences such as economics, population makeup, and behaviors, we found that places in the United States where the climate has changed more since the 1970s have a higher percentage of people with heart disease than more climate‐stable areas. Places with greater temperature change had the largest differences, but there were also differences associated with changes in sunlight, rain, pressure, and wind. Warming winters were associated with lower heart disease, but this was outweighed by heat increases in other seasons that were associated with higher heart disease. We cannot prove that climate change caused higher heart disease rates, but we accounted for many other potential causes. More research is needed and the possible large and costly health impacts are important to consider when deciding how much to limit future climate change.

## Key Points

- We found that areas in the United States with a higher extent of climate change since the 1970s have higher rates of cardiovascular disease
- Heat increase was associated with the largest differences in cardiovascular disease rates, but other climate changes were also associated
- Annual daytime heat increase was more associated with cardiovascular disease prevalence than increases in heat waves or summer heat alone

## 1 Introduction

The impacts of climate change on human health are increasing in scope and severity (M. L. Bell et al., 2024). The potential population health impacts of climate change on cardiovascular diseases (CVD), the leading cause of death worldwide, are particularly important (Khraishah et al., 2022). While exposure to extreme heat imparts extensive risk, CVD is also particularly sensitive to social, behavioral, and environmental conditions that are affected by climate change (Bhatnagar, 2017; Khraishah et al., 2022; Ragavan et al., 2020; Zafeiratou et al., 2021). From healthcare and productivity loss, the estimated financial costs of cardiovascular disease (CVD) in the United States (U.S.) alone exceeds $250 billion per year (CDC, 2025). Despite a strong downward trend in CVD rates since 1970, representing one of the greatest public health achievements over recent decades, CVD prevalence and mortality rates have recently begun to rise (Woodruff et al., 2024).

The immediate effects of extreme heat caused by climate change on near‐term cardiovascular hospitalizations and mortality are well‐documented (Anderson & Bell, 2009; M. L. Bell et al., 2024; Donaldson et al., 2003). Yet, the long‐term impacts of heat exposure on cardiovascular health are not well understood and may exceed the burden of acute health outcomes (Zafeiratou et al., 2021). Most studies examining the cardiovascular effects of extreme heat compare short‐term exposures across regions or specific populations (Liu et al., 2022). However, populations experiencing larger increases in the frequency and severity of extreme heat and other climate stressors may be less prepared and acclimatized than those experiencing equivalent but consistent heat patterns (Hanna & Tait, 2015; Zacharias et al., 2015). Few past studies have directly examined the extent to which long‐term climate change or location‐based deviation from historical baselines, often referred to as climate anomalies, may impact health. Of these, across 17 European countries, there was higher mortality during a heat wave in places experiencing greater change from historical human thermal comfort index but otherwise equivalent heat exposure (Di Napoli et al., 2018). In New England, a 1°C rise in summer mean air temperatures was associated with 1% higher mortality, whereas a 1°C increase in winter mean air temperatures was associated with a 0.6% decrease in mortality (Shi et al., 2015). In pooled 3 U.S. nationwide cohorts with approximately 30 years of observations of 276,618 participants, 1.3°C higher summer air temperatures compared with the previous 5‐year summer average were associated with 3% higher CVD incidence among those <65 years of age, whereas 2.7°C higher winter air temperatures were associated with 9% lower CVD incidence (Klompmaker et al., 2025).

Location‐based changes in climate beyond heat can also affect cardiovascular health at the individual and collective level through precipitation, sunlight, pressure, and other climatic conditions. Changes in rainfall patterns can affect overall health and CVD risk through increased exposure to mold, waterborne and vector‐borne infectious diseases, and affect heat exposure due to humidity (Acero et al., 2024; Baker et al., 2022; D’Amato et al., 2020; Fouque & Reeder, 2019). Sunlight is necessary for vitamin D synthesis and regulates circadian rhythm, but also amplifies heat exposure (Holick, 2004; Pauley, 2004; Zhao et al., 2021). Low atmospheric pressure has been reported as an independent predictor of higher stroke incidence (Morabito et al., 2011). Wind patterns affect cooling, humidity, and air pollution exposures (Pantusheva et al., 2022; Pérez et al., 2020). The combination of multiple climate changes may also lengthen and intensify exposure to allergenic pollens (Anenberg et al., 2017; D’Amato et al., 2020).

Climate change over decadal scales also affects all domains of social determinants of health, well beyond direct exposure to heat and other climate stressors (Ragavan et al., 2020; Salas, 2022). Beyond extreme heat, moderate but uncomfortable weather conditions can dissuade active and public transportation, thereby reducing physical activity and access to facilities and services (Baobeid et al., 2021). Changing climate patterns affect social and community contexts of social cohesion and mental health, while also limiting physical activity in summer with the opposite during cooler seasons (Berry et al., 2010; Cherng et al., 2019; Obradovich & Fowler, 2017). Economic opportunity is harmed by infrastructure damage, productivity loss, decreased access, agricultural loss, supply chain disruption, and climate‐driven migration (Moser & Hart, 2015; Tol, 2009). Neighborhood built environments can be affected by disasters, biodiversity and greenspace loss, and increased air pollution (Orsetti et al., 2022). Despite such extensive evidence that demonstrates widespread effects of climate change on social and environmental determinants of health, the resulting cumulative effects on population health are not well understood. Importantly, it is likely that many of these indirect health impacts of long‐term climate change may exponentially increase as modern climate conditions progressively surpass the boundaries of historical ranges and resilience capacity (Ebi & Hess, 2020; Pörtner et al., 2022).

The ecological association between the location‐based extent of climate change and the population burden of CVD in the U.S. is not known. To address this fundamental knowledge gap, our primary objective for this study was to measure the association between climate change and cardiovascular disease across the continental U.S. We used the term “anomalies” when referring to location‐based changes in climate metrics from a historic base period and recent time frame. While our study does not directly attribute climate change to health outcomes (Stott et al., 2016) collectively considered these anomalies provide a much better representation of the extent and characteristics of location‐based climate changes than any previously published primary analysis of associations between climate change and health. Our secondary objective was to evaluate the characteristics of changes in climate that were most strongly associated and the temporal characteristics of associations. For these objectives, we compiled spatially resolved observations to holistically characterize climate from 1970 through 2022. We evaluated location‐based associations between long‐term climate changes and the prevalence of cardiovascular disease, while controlling for local social and environmental factors, across the continental U.S.

## 2 Methods

### 2.1 Climate Data and Change Calculations

To assess long‐term location‐based changes in climate, independent of localized landcover changes, we first collected climate observations from the European Union Copernicus program ERA5‐Land climate metrics (Table S12 in Supporting Information S1) from 1970 through 2022 (Gorelick et al., 2017). We included 14 climate metrics that entail climate components of temperature, humidity, rainfall, wind, sunlight, and surface pressure. Available at ∼11 km spatial resolution, this ERA5‐Land data set for North America is highly consistent with other historical climate data sets and reflects relatively stable global surface temperatures until the late 1970s (B. Bell et al., 2021). To further mitigate the influence of localized landcover features and their spillover effects in our climate change observation assessment, we spatially aggregated all metrics to climate division spatial units developed by the U.S. National Oceanic and Atmospheric Administration (NOAA). These climate division spatial units are spatially optimized for internally‐consistent temperature and rainfall patterns (Guttman & Quayle, 1996). With a mean area of 22,650 km^2^ across 344 divisions in the U.S., climate divisions are sufficiently large to take a small‐region mean of climate measures with relatively low influence from localized urban landcover. Due to the potential for large intra‐day variation in temperature and humidity to skew heat index calculations based on daily mean data calculations, we calculated heat index from ERA5‐Land temperature and dew point data on an hourly basis (Muñoz Sabater, 2019) and aggregated to daily and monthly temporal scales. For other metrics, where hourly calculations were not necessary for reliable measurement, we collected ERA5‐Land metrics for climate divisions with monthly temporal resolution. We used Google Earth Engine for all ERA‐5 climate data collection.

From the compiled climate metrics, we calculated long‐term mean climate anomaly values as the change between historical and recent time frames. To establish a temporal range from which to calculate anomalies, we selected 1970–1979 as a base period to compile climate observations immediately prior to the onset of progressive global climate changes (B. Bell et al., 2021; Santer et al., 2013), while also preventing misclassification from earlier time frames. We selected base and modern periods of 10 years to account for short‐term cyclical climate variability while preventing additional measurement dilution of the extent of change that would be introduced by taking the mean of longer time spans.

We calculated the mean anomaly size by subtracting the mean values of a historical reference base period (1970–1979) from the recent (2013–2022) climate time frame mean. From hourly heat index values, we compiled temporal subclassifications of heat index that include anomaly values specific to seasons, daytime and nighttime heat, heat variability, duration of extreme heat, and heat wave frequency and intensity. For sensitivity analyses, we calculated alternate baseline and recent time period bins of 20 years (i.e., 1970–1989, 2003–2022) as well as the mean of interim decades as well as more recent 10‐year base periods (i.e., 1980–1989, 1990–1999, and 2000–2009). We then assigned the climate division mean anomaly values to census tracts overlapping each climate division.

### 2.2 Health Outcomes Data

To account for the extensive variability of confounding factors (e.g., age, socioeconomics, behaviors) at larger spatial scales, we examined yearly cross‐sectional census tract (2010 boundaries) level estimates. Our primary outcome measures were the 2020–2022 yearly mean prevalence of adults living with coronary heart disease (CHD) and past stroke. We collected prevalence estimates, quantified by the CDC PLACES Project, for 72,337 tracts across the contiguous U.S. that encompass 90.28% of the total U.S. population (Greenlund et al., 2022). The CDC PLACES disease prevalence estimates are derived from small area estimate methods, primarily based on data from the Behavioral Risk Factor Surveillance System survey of over 400,000 U.S. adults per year (Greenlund et al., 2022). This small area estimation approach accounts for rate instability and various forms of survey and response bias found in BRFSS raw prevalence rates (Greenlund et al., 2022; Zhang et al., 2014). This approach has been previously validated nationwide for county‐level estimates (Zhang et al., 2014). While tract‐level estimates have not been validated nationwide due to lack of data availability, smaller‐scale validation studies have found high correlation between sub‐county small area estimates and prevalence measured through electronic health records (Chen et al., 2022). To account for potential residual estimate instability and intermittent missing data, we calculated the tract‐level mean of available CDC PLACES yearly prevalence and covariate estimates for the years 2020–2022.

### 2.3 Covariate Data

To account for physical and social characteristics that may covary with spatial patterns of climate anomalies (Spangler & Wellenius, 2020) we incorporated data from three nationwide tract‐level data sets: (a) The CDC PLACES project for health risk factors (Greenlund et al., 2022), estimates averaged for 2020–2022; (b) The CDC Agency for Toxic Substances and Disease Registry/Geospatial Research, Analysis, and Services Program ‐ Social Vulnerability Index (SVI) (Flanagan et al., 2018), 2018 estimates for social vulnerability and socioeconomics; and (c) The ESRI Climate Resilience Index data set 2023 estimates for impervious surface cover (Tools for Building Community Climate Resilience, 2024). To account for the potential of urban growth to affect both climate anomalies and health impacts of migration, we calculated the long‐term change in urban landcover. For this, we downloaded historical urban landcover estimates from the World Climate Change Research Programme Land‐use Harmonization data set (Eyring et al., 2016). To calculate change in urbanization, we aggregated estimates of the percent of urban area cover to climate divisions and subtracted the percent of urban landcover in 1971 from the percentage of urban landcover in 2018. All model covariates, except for change in urban landcover, were contemporaneous with outcome prevalence estimates in order to account for the many modern influences of population characteristics that affect CVD trends independent of climate change.

### 2.4 Statistical Analysis

From the initial set of 71,830 continental U.S. census tracks, we excluded tracts from the analysis that were missing necessary data of CHD prevalence, smoking prevalence, prevalence of those receiving annual checkups, socioeconomic status, impervious surface cover, and urban landcover change. In total, we excluded 163 tracts due to missing data, with 71,667 tracts remaining. With remaining tracts as the unit of analysis, we developed a multivariate linear mixed effects model to test associations between each climate anomaly metric (calculated at the climate division level and assigned to nested tracts) and outcome of CDC PLACES CHD and Stroke prevalence estimates, mean of years 2020–2022 estimates, at the tract level. As existing evidence indicates that heat exposure is a particularly important influence of climate change on CVD (Khraishah et al., 2022; Liu et al., 2022; Zafeiratou et al., 2021), we developed our models using heat index mean anomaly as the primary independent variable and CHD as the dependent variable. In a systematic approach to model selection, we iteratively tested potential model permutations of demographic, environmental, and population health characteristics to identify an initial best‐performing model based on *R* ^2^, RMSE, AIC, and BIC model fit statistics. To minimize residual confounding and spatial autocorrelation, we tested additional permutations of random intercepts, mixed effects terms, and spatially lagged outcome variables (supplementary methods). We excluded unstable models and identified our final model based on model performance. As a sensitivity analysis, we compared results between each major step of model development to evaluate coherence and stability (Table S3 in Supporting Information S1). When using stroke as the dependent variable in the final model, we found improved but similar model fit statistics.

We applied the final model to each climate anomaly metric and outcome permutation to evaluate associations of individual climate anomaly measures with CHD and stroke. To evaluate the extent to which observed change in each climate metric from the base to recent time periods were associated with CHD and stroke, we calculated the “effect size of anomaly” as the model per‐unit coefficient effect size per 1 unit difference multiplied by the national mean metric change over time (anomaly value) for the respective metric. To comprehensively evaluate the association between climate change and outcomes, beyond associations with individual anomaly metrics, we concurrently included all noncollinear (Pearson's *R* ^2^ correlation of <0.4 with other metrics and model variance inflation factor <10) anomaly metrics into the model. To accurately represent the true population‐level effect sizes of observed associations, we weighted these results by tract population to report a population‐weighted mean effect size of anomalies in this comprehensive climate change model. We confirmed improved model fit of this multiple metric model against other fit tests used in the single‐metric model development approach.

### 2.5 Sensitivity Analyses

To ensure that results were robust to cyclical climate variability, we conducted a parallel sensitivity analysis using a 20‐year baseline (1970–1989) and 20‐year recent (2003–2022) timeframe. We also evaluated associations between anomalies and health outcomes with additional anomaly baseline time frames of 1980–1989, 1990–1999, and 2000–2009. To evaluate the influence of migration on overall results, we compared the results of our primary model stratified by counties in the top and bottom 50th percentiles of in and out migration rate. We tested the comprehensive model sensitivity to potential anomaly metric collinearity by comparing alternate metric exclusion criteria of *R* ^2^ < 0.8, <0.6, and <0.2. As a sensitivity analysis of associations found in our models, including multiple anomaly metrics, we conducted a principal component analysis of all climate anomaly metrics and replaced climate anomaly metrics in the model with component factor scores. While a subsequent study is needed to fully explore differences in sensitivity across people‐ and location‐based characteristics, we used our primary model to test associations among tract stratifications of highest and lowest 50th percentile of both SES and modern temperature. We also compared tracts classified as metropolitan core areas (*n* = 51,506), based on rural‐urban commuting area codes (RUCA), with less urbanized areas (*n* = 20,324). We conducted all statistical analyses in R (v.4.2.2–4.5.0) statistical software. Detailed methods are described in Appendix in Supporting Information S1.

## 3 Results

### 3.1 Climate Anomalies

**Figure 1:**
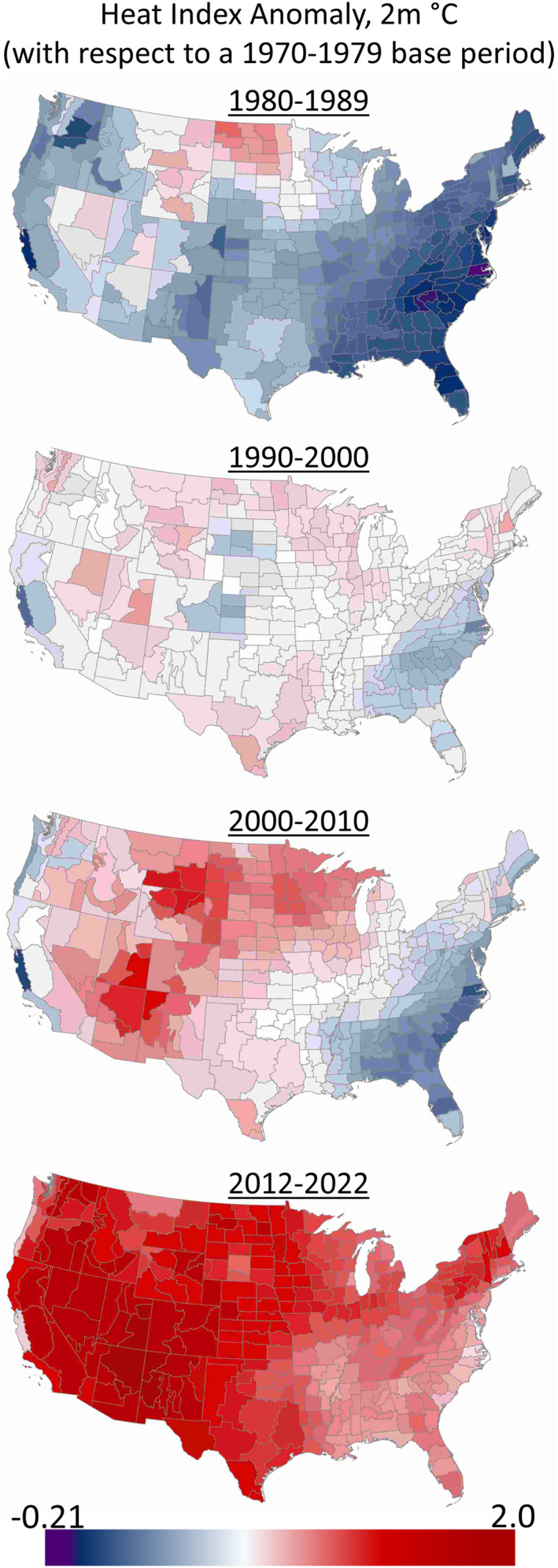
Decadal heat index anomalies. Change in mean heat index from a 1970–1979 base period time frame to the mean of following decades, aggregated to the climate division scale. Continuous color scale bins by consistent equal intervals.

For the years 1970 through 2022, we observed progressive and regionally varied changes across a wide suite of climate metrics, including heat index (Figure 1). Across the continental U.S., mean climate anomaly metrics reflected an increase in mean air temperature (+1.05°C), heat index (+1.11°C), dew point (+0.71°C), northward component of wind speed (+0.07 m/s), absorbed sunlight (+1.29 × 10^5^ J/m^2^), net thermal radiation (+4.38 × 10^6^ J/m^2^), sensible heat flux (+2.35 × 10^6^ J/m^2^), surface pressure (+6.62 × 10 Pa), and total precipitation (+5.01 × 10^−3^ m/year). Annual mean climate anomaly metrics reflected a decrease in mean relative humidity (−1.00%), eastward component of wind (−0.01 m/s), surface latent heat flux (−7.18 × 10^6^ J/m^2^), total evaporation (−2.88 × 10^−3^ m of equivalent water), transpiration (−9.29 × 10^−5^ m of equivalent water), and sunlight (−2.87 × 10^6^ J/m^2^). The geographic trend of anomaly metrics was largely distinct for metrics that were not closely interrelated (Figure S1 in Supporting Information S1).

### 3.2 Location‐Based Associations Between Individual Climate Anomaly Metrics and Health Outcomes

Resulting from our model development and fitting approach, our primary random intercepts linear model, with U.S. census tracts as the unit of analysis, included covariates of socioeconomic status (SES), smoking prevalence, obesity prevalence, proportion of residents above age 65, prevalence of those receiving a yearly medical checkup, percent impervious surface cover, change in impervious cover from the climate base to modern period, and recent (2013–2022) annual temperature mean. This model also included county‐level random intercepts to account for potential residual variation within the respective county. After excluding tracts missing necessary data, the model encompassed 71,667 census tracts containing 99.85% of the continental U.S. population.

**Figure 2:**
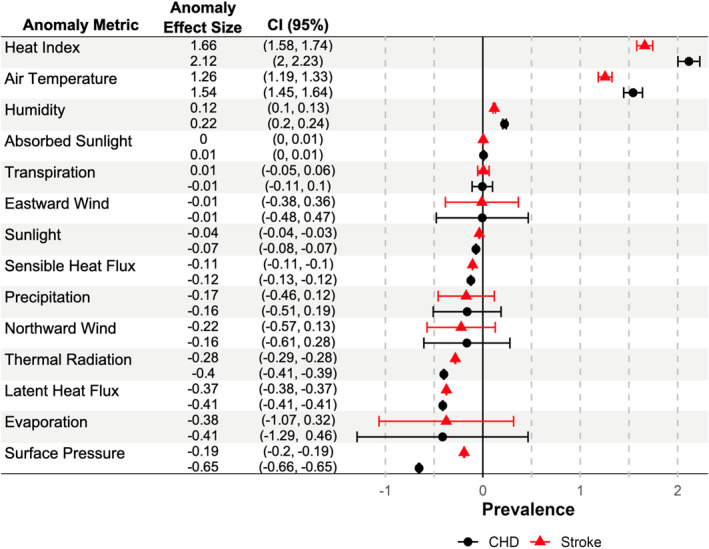
Association of coronary heart disease and stroke with climate anomalies in single‐metric models. Anomaly metrics calculated as the difference between a base period of 1970–1979 and 2013–2022 in separate models. Anomaly effect size represents the per‐anomaly‐unit population prevalence effect size of associations of each metric multiplied by the mean metric anomaly value in respective units (*n* = 71,667). CI = 95% confidence interval.

We found that the overall location‐based extent of numerous observed changes in climate from a 1970–1979 base to a 2013–2022 recent mean was significantly associated with CHD and stroke prevalence (Figure 2). We found that heat index anomaly was most strongly associated with higher population prevalence of CHD (2.12% CI: 2.00%, 2.23%) and stroke (1.66% CI: 1.58%, 1.74%). We found comparable but smaller associations for air temperature anomaly (CHD: 1.54% CI: 1.45%, 1.64%, Stroke: 1.26% CI: 1.19%, 1.33%). The effect of other anomalies in independent models varied widely in size and direction. There were smaller positive associations between CHD and stroke with relative humidity anomaly. We observed significant inverse associations of CHD and stroke with anomalies of latent heat flux, thermal radiation, sensible heat flux, surface pressure, and sunlight.

### 3.3 Comprehensive Climate Anomaly Model Results

When including all anomaly metrics that were not highly correlated in a single model to account for concurrent changes in climate components, we observed similar but attenuated results when compared with individual‐anomaly models (Table 1). Heat index and surface pressure anomalies had large associations in mixed directions with population prevalence of both CHD (heat index: 1.63% CI: 1.52%, 1.74%, pressure: −0.76% CI: −0.77%, −0.75%) and stroke (heat index: 1.59% CI: 1.51%, 1.67%, pressure: −0.34% CI: −0.34%, −0.33%). This corresponds to 1.46% higher population prevalence of CHD and 1.42% higher prevalence of stroke per 1°C increase in heat index from the baseline to modern time frame. The extent of other anomalies was associated with a −0.17%–0.01% difference in CHD and −0.29%–0.08% difference in stroke prevalence. The sum of these differences, reflecting cumulative effect size, was 0.59% for CHD (9.84% relative prevalence) and 0.88% for stroke (27.88% relative prevalence). We found these effect sizes to be consistent with our parallel sensitivity analyses of *R* ^2^ exclusion points of <0.2, <0.6, and <0.8 (Table S5 in Supporting Information S1).

**Table 1:** Comprehensive Association of Climate Anomalies With Coronary Heart Disease and Stroke

Anomaly metric (scale factor)	Anomaly size	CHD			Stroke		
		Effect size per unit change (CI)	Anomaly effect size (CI)	Relative prevalence	Effect size per unit change (CI)	Anomaly effect size (CI)	Relative prevalence
Heat index anomaly	1.12	1.46 (1.34, 1.57)	1.63 (1.52, 1.74)	26.93	1.42 (1.35, 1.50)	1.59 (1.51, 1.67)	50.31
Eastward wind anomaly	−0.01	−1.41 (−1.80, −1.03)	0.01 (−0.37, 0.40)	0.24	−0.70 (−0.99, −0.41)	0.01 (−0.28, 0.30)	0.23
Northward wind anomaly	0.06	−1.67 (−2.02, −1.32)	−0.11 (−0.46, 0.24)	−1.79	−2.67 (−2.94, −2.41)	−0.17 (−0.44, 0.09)	−5.47
Evaporation anomaly (×10)	−0.03	6.00 (4.98, 7.03)	−0.17 (−1.20, 0.85)	−2.82	10.05 (9.33, 10.77)	−0.29 (−1.00, 0.43)	−9.03
Surface pressure anomaly (×10^−^1)	6.53	−0.12 (−0.12, −0.11)	−0.76 (−0.77, −0.75)	−12.53	−0.05 (−0.06, −0.05)	−0.34 (−0.51, −0.50)	−10.70
Transpiration anomaly (*10^3^)	−0.09	−0.06 (−0.16, 0.04)	0.01 (−0.09, 0.10)	−0.08	0.04 (−0.02, 0.10)	0.00 (−0.06, 0.06)	−0.11
Sunlight anomaly (×10^−^6)	−3.05	0.01 (0.00, 0.01)	−0.02 (−0.02, −0.01)	−0.27	−0.03 (−0.03, −0.02)	0.08 (0.08, 0.09)	2.65
Sum effect:		CHD:	0.59	9.84	Stroke:	0.88	27.88

*Note.* Results of models including all annual anomaly metrics with *R* ^2^ < 0.4 with all other metrics and model VIF <10. Effect size per unit change represents the model coefficient effect size per 1 unit of the respective anomaly. Anomaly effect size represents the population‐weighted model coefficient effect size of each metric multiplied by the metric anomaly size for persons aged 18 and older in respective units, resulting in units of percent population prevalence. Relative prevalence represents the anomaly effect size as a percentage of the outcome population prevalence. CI = 95% confidence interval.

### 3.4 Associations by Time of Day, Season, and Extreme Temperature Anomaly

**Figure 3:**
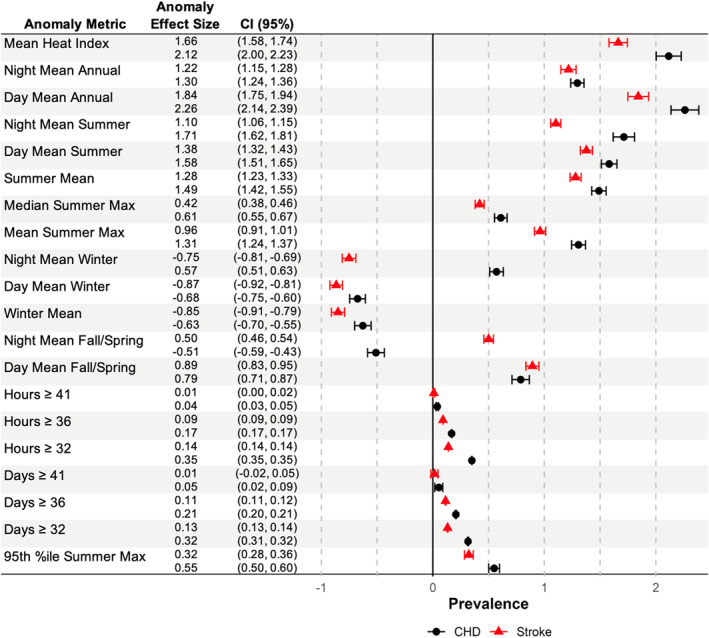
Association of heat index temporal stratification anomalies with coronary heart disease and stroke. Comparison of each temporally delineated heat index anomaly metric between a baseline of 1970–1979 and 2013–2022 in separate models. The anomaly effect size was computed as the model coefficient multiplied by the mean metric anomaly size (*n* = 71,667). All heat index measurements are in units of Celsius. CI = 95% confidence interval.

We observed that annual mean daytime heat index anomaly had similar but slightly larger effect sizes of positive associations with CHD and stroke (Figure 3). Anomalies of summer mean heat index, annual mean nighttime heat index, and summer mean nighttime heat index had notably larger associations than other temporal stratifications. In contrast, we found that anomalies of winter 24 hr and winter daytime mean heat index had negative associations with prevalence of CHD and stroke. Winter nighttime heat index anomaly was positively associated with CHD and negatively associated with stroke. Fall and spring daytime heat index anomaly was positively associated with CHD and stroke, whereas fall and spring nighttime anomaly was positively associated with stroke and negatively associated with CHD. Associations between the anomaly of total number of days and hours reaching above a given heat index were stronger at 32°C than anomalies of higher temperatures. Anomalies of the number of heat waves and the variability of heat were comparatively small (Figure S6 in Supporting Information S1).

### 3.5 Sensitivity Analyses

In sensitivity analyses, we observed that interim models that include health and demographic adjustments in our model fitting process were consistently stable, with results similar to the final model (Table S3 in Supporting Information S1). When we compared permutations of spatial scales of model mixed effects terms and time frames of temperature adjustments, we found the best model fit to be with modern temperatures and a county level mixed effects term (Table S3 in Supporting Information S1). When comparing permutations of anomalies calculated with more recent anomaly base periods, we observed largely consistent directionality but inconsistent extents of associations that generally trended toward attenuated associations with more recent anomaly base periods (Figure S5 in Supporting Information S1). Because effect sizes are calculated by the model coefficient multiplied by the mean size of each respective anomaly, these attenuated associations were largely due to lower calculated anomaly values from temporal ranges that do not capture the full extent of climate change after the 1970's. When assessing the final random intercepts model with a 20‐year base period (1970–1989) and 20‐year recent time period (2003–2022), we again observed similar but attenuated associations (Table S4 in Supporting Information S1). These attenuated associations were again largely due to lower anomaly values, in this case resulting from the 20‐year baseline mean definition. These 20‐year mean values effectively eliminate accounting of changes occurring from 1970 through 1989 and attenuate more recent changes in the anomaly calculation by considering an additional 10 earlier years in the recent comparison time frame. With the same model as the primary analyses of individual anomaly metrics, with results presented in Figure 2, we repeated the analysis among tracts within counties with the lowest rates of migration (Table S6 in Supporting Information S1) and highest rates of migration (Table S7 in Supporting Information S1). We found attenuated association effect sizes among low migration counties and increased effect sizes in high migration counties, with consistent trends in comparable effect sizes between metrics. Upon stratifying tracts, we found similar associations between high and low SES tracts, moderately stronger effects in warmer areas, and substantially larger effect sizes outside of tracts in dense urban core areas (Table S8 in Supporting Information S1). In a principal component sensitivity analysis, we found that 62.8% of the variability of all anomaly metrics was attributable to a single main component, with loadings distributed across diverse anomaly metrics (Table S9 in Supporting Information S1). Other influential principal components generally reflected anomalies in sunlight and pressure (10.7%), lower wind (7.8%), and increased heat more specifically (5.9%). To display spatial distribution of these clustered patterns of changes in climate, we mapped principal components 1–6 (Figure S8 in Supporting Information S1) and found widely differing spatial distributions of each principal component. We then used principal component analysis factor scores in the primary model in place of anomaly metrics. While this cannot directly link individual anomaly metrics to outcome effect sizes, we observed results that were consistent in direction and relative proportion with corresponding metrics in our primary random intercepts models (Table S10 in Supporting Information S1).

## 4 Discussion

We observed a large but complex positive location‐based association between climate anomalies, a measure of location‐based climate change, and the prevalence of CHD and stroke across the continental U.S. We found the strongest positive associations for CHD and stroke with annual heat index anomalies. Yet, heat index and other climate anomalies were highly intertwined and showed mixed directionality in associations with stroke and CHD. When considered collectively, there was an overall association between the extent of location‐based climate change anomalies and higher prevalence of CHD and stroke, corresponding respectively to 9.8% and 27.9% higher relative prevalence. Based on temporal stratifications, the overall association of heat index anomalies with CHD and stroke were primarily due to daytime and summer anomalies and were partially mitigated by warmer winters. Taken together, these associations indicate that the overall association of location‐based climate change with CHD and stroke was largely due to increasing heat but was affected by climate anomalies well beyond increases in extreme heat.

The results of our sensitivity analyses confirm that the primary analysis results are robust to varied temporal anomaly definitions, modeling approaches, and concurrently occurring changes in many aspects of climate beyond heat. We observed generally similar associations when stratifying the analysis by high versus low SES and modern warmest versus coolest tracts. When stratified by urbanicity, we observed substantially larger effects in tracts outside of urban core areas. Our analysis cannot evaluate why the associations between climate anomalies and CVD are larger in less urban areas, but this finding may be related to differences in resilience infrastructure, access to relevant services, and a higher distribution of dense urban areas along coastlines with generally more stable temperature patterns than interior areas. We observed comparable associations between our comprehensive climate change model of all non‐colinear climate anomaly metrics and the climate anomaly metric groupings in our principal component sensitivity analyses. This holistic evaluation of multiple climate anomaly metrics may help to elucidate the intertwined nature of health‐relevant changes in climate that extend beyond heat to climate components such as sunlight, rainfall, wind, and surface pressure. Further, these findings demonstrate that the use of a single anomaly metric to assess associations between climate change and health likely reflects the effects of many concurrent changes in climate.

Recently, the health effects of non‐optimal temperatures, well below commonly accepted thresholds for extreme heat, have come into focus (Gasparrini et al., 2015; Tobías et al., 2021). These relatively low “optimum” temperatures for health (i.e., 13.0–26.5°C, largely depending on long‐term adaptation) (Tobías et al., 2021) align with our observation that annual temperature increases were associated with larger differences in CVD than increases in summer alone, or the frequency and extent of extreme heat. Consistent with established literature regarding the health impacts of cold temperatures, we found that a larger extent of winter temperature increase was associated with lower rates of CVD (Gasparrini et al., 2015; Zafeiratou et al., 2021). However, the effect size of our observed inverse winter association was eclipsed by the detrimental effects of higher temperatures in other seasons. While few past evaluations have evaluated the potential effects of atmospheric pressure on health, low atmospheric pressure has previously been reported as an independent predictor of stroke incidence (Morabito et al., 2011). This past finding aligns with our observation of associations between the extent of increased surface pressure and lower CVD prevalence. However, pressure increase anomaly values demonstrated a high co‐occurrence with increases in sunlight, which may also be responsible for the observed associations through increased vitamin D synthesis, physical activity, circadian alignment, and mental health, which have previously been linked to increased sunlight (Holick, 2004; Mead, 2008).

To our knowledge, no prior analysis has quantified the impacts of comprehensively measured long‐term climate changes on CVD. However, domain‐specific influences of climate change on the social and environmental determinants of global CVD and other disease burdens have been extensively documented (Berry et al., 2010; Obradovich & Fowler, 2017; Ragavan et al., 2020; Salas, 2022). These include air pollution exposure, physical activity and other health behaviors (Acero et al., 2024; Baobeid et al., 2021; Obradovich & Fowler, 2017), nutrition (Fanzo et al., 2018), infectious disease (Baker et al., 2022; Fouque & Reeder, 2019), mental health (Berry et al., 2010), and social determinants of health (Baobeid et al., 2021; Cherng et al., 2019; Orsetti et al., 2022; Ragavan et al., 2020; Tol, 2009). Disruption to existing climate‐adapted behaviors and systems may impart additional health impacts (Ebi et al., 2021; Ragavan et al., 2020; Winn et al., 2011).

A key strength of our investigation is the evaluation of spatiotemporally consistent long‐running historical climate data with tract‐level CHD and stroke prevalence estimates that represent 99.85% of the continental U.S. population. For the first time, our work provides a first‐order assessment of changes in a variety of weather measures (anomalies) over a long period of time and their location‐based association with CVD prevalence at the end of this time frame. This study advances beyond past research of associations between short‐term temporal associations between climate exposures, not attributed to climate change, and spatiotemporally‐linked health impacts. We observed sensitivity analyses results that were consistent with the primary observations and contribute additional context. Our evaluation is bolstered by accounting for urbanicity change, sociodemographics, and profiles of climate change through co‐occurring climate anomaly metrics. For example, our principal components sensitivity analysis of anomalies revealed that long‐term climate change is characterized by spatiotemporally inconsistent covarying changes in climate systems, with wide‐ranging implications to contextualize findings from studies that evaluate associations between a single metric of climate change and health outcomes. A notable strength of our holistic approach, climate observations, and ecological health associations is the ability to serve as a rigorous foundation for future longitudinal study across convergent fields of health, attribution science, mitigation and adaptation policy, and economics.

The analysis is limited by the extensive endogenous nature of climate anomalies, the likelihood of some residual confounding, lack of accounting for air conditioning use and other mitigating factors, and the inability to evaluate potential causative effects and pathways. Our approaches to include non‐colinear metrics and principal components analysis based mixture analysis are a step to address complex patterns of location‐based climate changes but cannot reliably assess the extent to which our observed associations may be effectively loaded onto, or distributed across, other co‐occurring anomaly metrics. Because of this, it is plausible that observed effect sizes may partially reflect influences of co‐occurring anomaly metrics in the total change profile. While internally‐consistent, products like ERA5‐Land have been shown to underestimate hot‐humid and dry air temperature extremes; thus, the extent of heat anomalies in our analyses may not fully account for the most extreme heat (Raymond et al., 2020; Verdin et al., 2020). The tract‐level prevalence estimates assessed as the study outcome are affected by corresponding CVD incidence and survival rates, both of which may also be influenced by climate change. As tract‐level CVD prevalence rates over long spans of time are not available, our analyses are strictly limited to an ecological evaluation of associations between the location‐based extent of climate anomalies and recent cross‐sectional prevalence estimates. While based on validated methodologies, these prevalence estimates are not an exact measure of population prevalence. We are unable to rule out potential ecological fallacies. Thus, the results should not be used as evidence to confirm or deny causative impacts of climate change on cardiovascular health and disease risk. However, existing literature demonstrates that climate change affects the fundamental drivers of global health, lending plausibility to the potential causative influences of climate change on CVD well beyond short‐term impacts. Finally, we note that while we define climate change based on long‐term changes in climate anomalies and find statistically significant associations, our study does not directly attribute anthropogenic climate change to health outcomes.

Our analysis of climate anomalies represents a novel approach toward evaluating the extent of location‐based associations between climate change on health. Future research is needed to address the limitations of this study and evaluate the potential wide‐ranging cumulative impacts of climate change on chronic diseases and society. The need for causal evaluation is of particular importance. Longitudinal analyses among cohorts with repeat observations across a wide geographic and temporal range (e.g., Nurses Health Study across the U.S., the Prospective Investigation into Cancer and Nutrition cohort across the European Union, and Gateway to Global Aging harmonized cohorts) could evaluate the consistency of results with our ecological evaluation (Colditz et al., 1997; Lee et al., 2021; Riboli & Kaaks, 1997). Such longitudinal cohort analyses could also evaluate at‐risk populations, the role of social determinants, and physiological mechanisms, with the potential to inform public health and medical practice and policy. Location‐based long‐term climate change analyses could also be used to evaluate long‐running health outcomes compilation data sets with high spatiotemporal resolution. Further research regarding the location‐based impacts of climate change on population health may further offer a new perspective on climate change attribution science. Beyond the direct implications of the health impacts of climate change, research is also needed to understand the full scope of potentially extensive economic costs resulting from healthcare costs and productivity loss. Given the $4.5 trillion in annual U.S. healthcare expenditures (CDC, 2024), any robust attribution of costs due to climate change could weigh heavily on financing considerations for climate change mitigation policies.

Our observation of a large and nuanced ecological association between location‐based climate anomalies and prevalence of CHD and stroke may, in part, reflect the nature and extent of climate change influences on CVD. These climate changes entailed deeply intertwined changes in aspects of climate with complex spatiotemporal patterns of long‐term change. Based on the preponderance of existing evidence, further changes in climate that are locked in due to accumulated and ongoing emissions may have profound health impacts for future generations (Mora et al., 2018). The need to holistically understand the effects of climate change on chronic disease, for medical practice and environmental health policy, is urgent. Yet, near‐term climate change mitigation policy is being formed in the absence of conclusive evidence and will likely determine whether cascading irreversible changes in Earth systems are triggered. Careful consideration of the precautionary principle is warranted regarding the potential permanent and nonlinear global health impacts of climate policy.

## Conflict of Interest

The authors declare no conflicts of interest relevant to this study.

## Supporting information

Supporting Information S1

## Data Availability

All raw data employed in this study were publicly available at the time of the study via cited data sources. We used Google Earth Engine, available at earthengine.google.com, for all ERA‐5 climate data collection. ERA‐5 data was developed by the European Union Copernicus program (Gorelick et al., 2017). Climate division spatial units were developed by the U.S. National Oceanic and Atmospheric Administration (Guttman & Quayle, 1996), with spatial data and supporting information available at www.ncei.noaa.gov/access/monitoring/dyk/us‐climate‐divisions through NOAA. The CDC PLACES project outcomes and risk factors data (Greenlund et al., 2022) are available at www.cdc.gov/places/tools/data‐portal.html through the CDC data portal. The CDC Agency for Toxic Substances and Disease Registry/Geospatial Research, Analysis, and Services Program ‐ SVI (Flanagan et al., 2018) data and documentation are available through the CDC SVI website (www.atsdr.cdc.gov/place‐health/php/svi/svi‐data‐documentation‐download.html). In the event that the original data portal for CDC data is no longer available, PLACES and SVI data are available through various data repositories, including the Harvard Dataverse. We accessed the ESRI Climate Resilience Index data set 2023 estimates for impervious surface cover (“Tools for Building Community Climate Resilience”) through ESRI ArcGIS Pro software. We downloaded historical urban landcover estimates from the World Climate Change Research Programme Land‐use Harmonization data set (Eyring et al., 2016) 1971 and 2018 layers from the Oak Ridge National Laboratory Distributed Active Archive Center for Biogeophysical Dynamics, and subsequently available through the NASA Earthdata Platform (Chini et al., 2014). We used ESRI ArcGIS pro software for spatial aggregation and spatial data linkages (www.esri.com/en‐us/arcgis/products/arcgis‐pro/overview). We conducted all statistical analyses with publicly available R (v.4.2.2–4.5.0) statistical software (https://www.r‐project.org/). We provide compiled ERA‐5 climate data and code for analysis contained in this manuscript on figshare (Yeager, 2026).
