# Supplementary material for: Association Between Observed Climate Change and Cardiovascular Disease in the United States
Source: Geohealth. 2026 Apr 13;10(4):e2025GH001588. doi: 10.1029/2025GH001588 (PMC13071862; doi:10.1029/2025GH001588)
Supplement: Supplementary file 1 — Supporting Information S1 [file GH2-10-e2025GH001588-s001.pdf]

GeoHealth

Supporting Information for:

Association between observed climate change and cardiovascular disease in the United States

Authors:

*Yeager, R.,[a,b,c] Tuholske, C.,[d,e] Browning, M.H.E.M.,[f] Mattingly, C.,[a,b] Olmsted, S.,[a,b] Ossola, A.,[g] Locke, D.H.[h]

[a]Division of Environmental Medicine, Department of Medicine, University of Louisville. Louisville, Kentucky, USA;
[b]Christina Lee Brown Envirome Institute, University of Louisville. Louisville, Kentucky, USA;
[c]Center for Integrative Environmental Health Sciences, University of Louisville. Louisville, Kentucky, USA;
[d]Department of Earth Sciences, Montana State University. Bozeman, Montana, USA;
[e]Geospatial Core Facility, Montana State University. Bozeman, Montana, USA;
[f]Department of Parks, Recreation, and Tourism Management, Clemson University. Clemson, South Carolina, USA;
[g]Department of Plant Sciences, University of California Davis. Davis, California, USA;
[h]United States Department of Agriculture Forest Service, Northern Research Station, Baltimore Field Station. Baltimore, Maryland, USA.

SUPPORTING INFORMATION

Supplemental Methods

Outcomes data 3
Climate data 3
Covariate data *6*
Statistical approach 6

Supplemental Figures

Figure S1: Maps of anomalies for ERA5-Land climate annual metrics 10
Figure S2: Maps of temporal strata of heat index anomalies 11
Figure S3: Maps of anomalies for temporal delineations of heat index 12
Figure S4: Maps of outcomes and covariate data 13
Figure S5: Comparison of baseline time definitions in associations between anomaly metrics and health outcomes 14
Figure S6: Association of heat wave and variability anomalies with CHD and stroke 15
Figure S7: Correlation matrices of anomaly metrics included in models 16
Figure S8: Spatial patterns of co-occurring climate anomalies 17

Supplemental Tables

Table S1: Demographic, health, and environmental variables by tertiles of the heat index anomaly from 1970-1979 and 2013-2022 18
Table S2: Health status and risk variables by tertiles of the heat index anomaly from 1970-1979 and 2013-2022 19
Table S3: Results from iterations of linear model development 20
Table S4: Sensitivity analysis of the association between health metrics and heat index with 20yr climate metric bins 21
Table S5: Sensitivity analysis of comprehensive model with permutations of R^2 exclusion criteria 22
Table S6: Sensitivity analysis of associations between anomalies and outcomes for areas with low migration 23
Table S7: Sensitivity analysis of associations between anomalies and outcomes for areas with high migration 24
Table S8. Stratification of associations between anomalies by demographic variables for CHD and stroke 25
Table S9: Principal component analysis loadings 26
Table S10: Random intercept model of principal components scores sensitivity analysis results 27
Table S11: Outcomes and covariates used in the analysis 28
Table S12: Climate variables used in the analysis 29

Supplemental References* *30

Supplemental Methods

Health outcomes data

The primary data sources for the modeling and estimation used to develop CDC PLACES prevalence estimates include the Behavioral Risk Factor Surveillance System (BRFSS) and American Community Survey (ACS) (Greenlund et al., 2022). The PLACES estimates were developed using a small area estimation multilevel modeling framework, with a multilevel regression and poststratification (Greenlund et al., 2022; Zhang et al., 2015). With this approach, prevalence estimates for each variable were developed at the census block level and then aggregated to the much larger census tract level to ensure reliable estimates (Greenlund et al., 2022; Zhang et al., 2015). These estimates have been internally and externally validated against health survey estimates, with high and consistent correlations between estimates and validation datasets (Pierannunzi et al., 2016; Wang et al., 2017; Zhang et al., 2015). The developers of the PLACES dataset caution that "some statistical collinearity is possible" for analyses of the dataset due to the modeling and estimation approach (Greenlund et al., 2022). Health outcomes of PLACES include prevalence of CHD, hypertension, stroke, chronic obstructive pulmonary disease, high cholesterol, diabetes, cancers, obesity, asthma, kidney disease, poor mental health, and depression – representing the majority of morbidity and mortality in the U.S. Risk factors reported by PLACES include smoking, binge drinking, physical activity, sufficient sleep, health insurance, and screening. We downloaded tract-level PLACES data for the years 2020, 2021, and 2022 from the CDC PLACES website and calculated the tract-level mean for these years.

Climate data

We collected hourly air and dew point temperature as well as monthly aggregated climate data from the ERA5-Land dataset (Muñoz Sabater, 2019). With Google Earth Engine (GEE), we averaged hourly raster-based air temperature and dew point data within the 344 climate divisions across the continental U.S. that were developed by the U.S. National Oceanic and Atmospheric Administration (NOAA) (Gorelick et al., 2017). All averaged hourly data from 1970 to 2022 was exported from GEE as comma-separated values (csv) files (Gorelick et al., 2017). Before calculating relative humidity and heat index, we checked for air temperature and dew point outlying data. Dew point temperatures that exceeded the record maximum air temperature or dropped below -68°C were set to NA and excluded from the analysis (Daly et al., 2015; National Oceanic and Atmospheric Administration, n.d.). We also excluded hourly air and dew point temperatures if the dew point temperature was greater than air temperature or the dew point temperature was more than 10° higher than the previous hour (Daly et al., 2015). A total of 2,138 hours (< 1%) were omitted. We then used air and dew point temperature to calculate hourly relative humidity (Daly et al., 2015; Williams et al., 2024).

To derive heat index, we used a series of air temperature-dependent equations recommended by the National Weather Service (NWS). First, we calculated heat index using the equation developed by R.G. Steadman (1979) and used this value if it met two conditions: 1) the air temperature was > 40°F; 2) the average of air temperature and Steadman's heat index was < 80°F (National Weather Service, n.d.; Steadman, 1979). The Rothfusz (1990) regression equation was used when the average was ≥ 80°F.National Weather Service, n.d.; Rothfusz, 1990). An adjustment to the Rothfusz heat index

was applied when the relative humidity was < 13% and the air temperature ranged from 80-112°F or when the relative humidity was > 85% and the air temperature ranged from 80-87°F (National Weather Service, n.d.). The final heat index value was converted to Celsius (Desai et al., 2021; Rajib et al., 2011). All computations were done in R (v 4.3.1) with the tidyverse package (Wickham et al., 2019).

We temporally aggregated all metric values to the climate division scale to calculate the difference between 1970-1979 and 2013-2022 for the: average heat index; number of days with a heat index above 32°C, 34°C, 38°C, and 41°C; number of hours with a heat index above 32°C, 34°C, 36°C, 38°C, and 41°C; average summer and winter heat index; variance of the daily, weekly, monthly, and yearly heat index; standard deviation in minimum and maximum summer and winter heat index temperatures; number of 4- and 7-day heat waves for a heat index above 32°C, 34°C, and 36°C; average fall/spring, summer winter, and annual nighttime heat index; average daytime fall/spring, summer, winter, and annual heat index; summer median and summer mean of daily maximum heat index; 95th percentile of summer maximum daily heat index; and the difference between the 95th percentile and median of daily maximum heat index. We then assigned this climate division value to overlapping census tracts.

All heat index anomaly variables were calculated as the difference between 1970-1979 mean and 2013-2022 mean of the following variables:

- Mean Heat Index: Average heat index
- Days ≥ 32: Number of days with a heat index above 32°C
- Days ≥ 34: Number of days with a heat index above 34°C
- Days ≥ 36: Number of days with a heat index above 36°C
- Days ≥ 38: Number of days with a heat index above 38°C
- Days ≥ 41: Number of days with a heat index above 41°C
- Hours ≥ 32: Number of hours with a heat index above 32°C
- Hours ≥ 34: Number of hours with a heat index above 34°C
- Hours ≥ 36: Number of hours with a heat index above 36°C
- Hours ≥ 38: Number of hours with a heat index above 38°C
- Hours ≥ 41: Number of hours with a heat index above 41°C
- SD HI Daily: Standard deviation of daily heat index
- SD HI Weekly: Standard deviation of weekly heat
- SD HI Monthly: Standard deviation of monthly heat index
- SD HI Yearly: Standard deviation of yearly heat index
- SD Min HI Summer: Standard deviation of summer daily minimum heat index
- SD Max HI Summer: Standard deviation of summer daily maximum heat index
- SD Min HI Winter: Standard deviation of winter daily minimum heat index
- SD Max HI Winter: Standard deviation of winter daily maximum heat index
- 4 days HI ≥ 32: Number of 4-day heat waves with heat index above 32°C

- 7 days HI ≥ 32: Number of 7-day heat waves with heat index above 32°C
- 4 days HI ≥ 34: Number of 4-day heat waves with heat index above 34°C
- 7 days HI ≥ 34: Number of 7-day heat waves with heat index above 34°C
- 4 days HI ≥ 36: Number of 4-day heat waves with heat index above 36°C
- 7 days HI ≥ 36: Number of 7-day heat waves with heat index above 36°C
- Night Mean HI Annual: Annual mean nighttime heat index
- Night Mean HI Fall/Spring: Mean nighttime heat index during fall/spring (October-November/March-May)
- Night Mean HI Summer: Mean nighttime heat index during summer (June-September)
- Night Mean HI Winter: Mean nighttime heat index during winter (December- February)
- Day Mean HI Annual: Annual mean daytime heat index annual
- Day Mean HI Fall/Spring: Mean daytime heat index during fall/spring (October-November/March-May)
- Day Mean HI Summer: Mean daytime heat index during summer (June-September)
- Day Mean HI Winter: Mean daytime heat index during winter (December- February)
- Summer Mean HI: Mean summer (June-September) heat index
- Winter Mean HI: Mean winter (December-February) heat index
- Mean Summer Max HI: Maximum daily heat index in summer (June-September)
- Median Summer Max HI: Median maximum daily heat index in summer (June-September)
- 95th %ile Summer Max HI: 95th percentile of maximum daily heat index in summer (June-September)
- 95th %ile vs Median Max HI Summer: Difference between the 95th percentile and the median maximum daily heat index in summer (June-September)

We similarly aggregated the monthly climate metrics to calculate average eastward component of wind, average northward component of wind, average total surface latent heat flux, average total absorbed sunlight, average total sunlight, average total net thermal radiation, total sensible heat flux, average of total evaporation, average surface pressure, average total precipitation, average total transpiration. All climate metric anomaly variables were calculated as the difference between 1970-1979 mean and 2013-2022 mean of the following variables:(Muñoz Sabater, 2019)

- Eastward Wind: average eastward 10m elevation wind
- Northward Wind: average northward 10m elevation wind
- Sunlight: average amount of solar radiation reaching the surface
- Evaporation: average amount of water evaporated from the surface
- Surface Pressure: average force of the atmosphere on land and water
- Precipitation: average liquid and frozen water that has fallen to the surface
- Transpiration: average amount of evaporation from vegetation transpiration
- Latent Heat Flux: average rate of energy transfer from water phase changes at the surface (evaporation/condensation; flux = transfer rate per unit area).

- Sensible Heat Flux: average heat transfer between surface and air carried by moving air, excluding moisture-related heat (flux = transfer rate per unit area).
- Absorbed Sunlight: average sunlight retained by the surface—incoming sunlight minus the portion reflected.
- Thermal Radiation: average net infrared heat at the surface—radiation from the air minus radiation emitted by the surface.

Covariate data

We collected socioeconomic and social vulnerability data at the tract level from the CDC Social Vulnerability Index (SVI). SVI includes nationwide tract-level data for age, race, and socioeconomic status (SES). As a holistic measure of SES, we used the socioeconomic sub-index score of the CDC Social Vulnerability Index (SVI) for analysis. This sub-index vulnerability score is compiled based on the tract-level proportion of residents with income below the 150% poverty level, unemployed, have an excess housing cost burden, do not have a high school diploma, and are without health insurance. Due to the need for matching tract data, we downloaded and used 2018 CDC SVI data, which was the last year SVI data was available that was compiled within 2010 census tract boundaries.

Covariates tested in model fit optimization and not retained in final models include a suite of tract-level health, demographic, and environmental characteristics. From PLACES, we tested asthma, depression, obesity, smoking, binge drinking, physical activity, low sleep, health insurance, routine checkup prevalence, mental health, and proportion of the population above age 65. From SVI, we tested the full SVI index score and sub-index scores for socioeconomic status, household characteristics, racial and ethnic minority status, and housing type and transportation. For environmental characteristics, we tested recent (2013-2022) air temperature and heat index compiled from ERA5-Land data, impervious surface cover, population density, tree canopy cover, and impervious surface cover change from 1971 to 2018.

Statistical approach

We excluded tract records that were missing necessary data for the analysis: CHD prevalence, smoking prevalence, prevalence of those receiving annual checkups, SVI-based SES, and percent impervious surface cover. We assessed population demographics by comparing tract-level tertiles of 1970-1979 to 2013-2022 heat index anomaly using the *tableone* package in R (v0.13.2) (Pollard et al., 2018; Yoshida et al., 2022). Given that Chi-sq and ANOVA tests with the large sample size would yield statistical significance with even minute differences between these tertiles, we did not test for significant differences between the tertiles.

With all included tracts for the continental U.S. (n=71,830, representing a population of 306,564,995 people), we iteratively developed a linear model to assess associations between each climate deviation metric and the prevalence of coronary heart disease. We tested permutations of model adjustments with the performance package in R (v.0.10.8) (Lüdecke et al., 2021, 2024). We first identified a preliminary adjusted linear regression model by comparing model

performance of potential adjustment permutations based on review of relevant literature and tract-level social and environmental differences between tracts with high and low heat index anomaly extents. In our initial model, we also included the percent of impervious surface cover change from 1971 to 2018 to account for large-scale urbanization that may also affect regional climate anomalies. We next tested permutations of mixed effect terms to improve model performance by accounting for local variation in the effect of model terms. We used a global Moran's-I test to evaluate spatial autocorrelation and tested permutations of spatially lagged outcome variables to account for spatial autocorrelation. With an initially optimized model, we iteratively tested and refined permutations model covariates, mixed effects terms, and spatially lagged outcome variables for model fit statistics and spatial autocorrelation. We excluded unstable models and identified our final model based on model performance.

Before conducting regression analyses, we scaled all climate metrics, excluding eastward wind and northward wind, by factors of 10 to the general magnitude of heat index anomaly values. Then, we formulated a mixed-effects linear model with the county geoidentifier serving as the random intercept variable and evaluated the association between each climate metric and two health outcomes: 1) the prevalence of coronary heart disease among adults ≥ 18 years old CHD, and 2) the prevalence of stroke among adults ≥ 18 years old. The anomaly effect size of each anomaly metric was calculated by multiplying the coefficient by the average anomaly size.

For the analysis concurrently considering multiple independent anomaly measures, we also calculated a population-weighted effect size to develop a directly applicable measure of the cumulative size of the association between climate change and prevalence of CVD and stroke. For this, we used the U.S. Census Bureau's American Community Survey population data from 2015-2019. We then multiplied the number of persons over age 18 by each climate anomaly metric value at the tract level, divided the dataset sum of the resulting value by the total dataset population, and multiplied the result by the respective metric model coefficients. All linear models were run in R using the lme4 package (v1.1-34) and the forest plots were generated with ggplot2 (v3.4.4) (Bates et al., 2015, 2024; Wickham et al., 2023). Other tables and figures were created with these packages as well as the multi-model inference (MuMIn, v1.47.5), car (v3.1-2), corrplot (v1.47.5), and Hmisc (v5.1-3) (Bartoń, 2024; Fox et al., 2023; Wei & Simko, 2021).

In final models, y_{ic} denotes the outcome; $A_{ik}$is the anomaly for climate metric k for tract i in county c; X_{ic} is the adjustment set defined in the main methods; α is the intercept; β_k is the percentage-point change in prevalence for a one-unit increase in A_{ik}; θ the covariate-coefficient vector; u_c the county random intercept; and ε_{ic} the residual (error term). We tested single-anomaly models (one anomaly at a time plus covariates). For presentation, we report an anomaly effect size when summarizing a single metric, specified as:

$$y_{ic} = \alpha + \beta_k A_{ik} + \theta' X_{ic} + u_c + \varepsilon_{ic}$$

For the comprehensive model, we included the subset S of anomaly metrics retained after collinearity screening using bivariate correlation (R^2<0.4) and variance inflation factors (VIF<10). Here, k indexes metrics and S denotes the retained set. To compute the weighted mean, we multiplied the number of adults (≥18 years) in each tract by the anomaly value, summed across tracts, and divided by the total adult population. Specified as:

$$y_{ic} = \alpha + \sum_{k \in S} \beta_k A_{ik} + \theta' X_{ic} + u_c + \varepsilon_{ic}$$

As a sensitivity analysis to test the potential influence of anomaly metric exclusion criteria and collinearity of included anomaly metrics, we adjusted the R^2 exclusion criteria for our primary comprehensive analysis, with results displayed in Table 1, from 0.4 to < 0.8, < 0.6, and < 0.2. We generated multiple candidate models based on these changes in criteria, retaining only those with the largest number of independent variables. From these, we selected variables with the lowest average R^2. We reran the model in Table 1 using the new sets of independent variables for R^2 < 0.8,< 0.6, and < 0.2 (Table S5).

To evaluate the potential effect of population migration on observed results, we compared the results of final models shown in Figure 2 with parallel analyses excluding high-migration tracts from counties using U.S. Internal Revenue Service county-level migration data. For this, we collected yearly in-migration and out-migration data for the years of 2011-2020, which have consistent migration recording and formatting. We calculated the total percentage of residents migrating into or out of each respective county during this time span, including domestic and international migration. Counties were defined as high migration if their percentage was above the 50th percentile for the specific migration type being analyzed (in- or out-migration), or low migration if below the 50th percentile. Counties were classified as having high combined in and out migration if both in-migration and out-migration were above their respective 50th percentiles, and low combined in and out migration if both were below. We repeated the previously described final models in Figure 2 using six restricted samples. These samples included tracts located within counties categorized as: low in-migration, low out-migration, and low combined migration (Table S6); and high in-migration, high out-migration, and high combined migration (Table S7).

To assess the potential effects of stratifying included tracts by key demographic variables, we compared the results of final models shown in Figure 2 with those obtained from models using tracts stratified by socioeconomic vulnerability, mean annual temperature from 2013-2022, and their 2010 Rural-Urban Commuting Area (RUCA) code. RUCA codes use measures of urbanicity, population density, and daily commuting patterns to classify census tracts along the urban-rural continuum. For our analysis, we stratified RUCA classifications by separating tracts coded as “urban core” (the most urbanized category) from all other tracts(“Rural-Urban Commuting Area Codes | Economic Research Service,” n.d.). For socioeconomic vulnerability and mean annual temperature from 2013-2022, tracts were stratified by defining

tracts as low if their value was below the 50th percentile, or high if above the 50th percentile. We repeated the previously described final models in Figure 2 using these stratified sets of census tracts (Table S8).

As a sensitivity analysis to reduce multicollinearity among our regression model with highly intertwined anomaly metrics, we conducted a principal component analysis (PCA) of all metrics in the comprehensive climate change mixed model and subsequent analogous mixed effects regression analysis with PCA factor scores. Before running the PCA, all variables were standardized to a mean of 0 and standard deviation of 1 to ensure comparability across metrics with very different scales of units of measurement. We performed the PCA analysis without factor rotation to maintain variance between components and facilitate concurrent regression modeling of multiple component scores. We then extracted the PCA analysis component loadings and substituted these loading for climate anomaly variables in the primary regression model used for climate anomaly values. The PCA component loadings generally reflected that each anomaly metric was highly intertwined with others, indicating that location-based climate anomalies and geospatial trends did not exist in isolation (Table S9). We found that the first component explained 62.8% of variation between climate anomalies and was made up of anomaly loadings from across varied aspects of climate change, without a single driving variable or highly correlated set of variables. However, the following 3 components respectively reflected anomalies of surface pressure, wind, and heat index more specifically. In our mixed effects model sensitivity analysis using PCA component scores in our final random intercepts model, the total anomaly size and direction of components aligned with results of the primary random intercepts model with multiple distinct anomaly metrics (Table S10).

Supplemental Figures

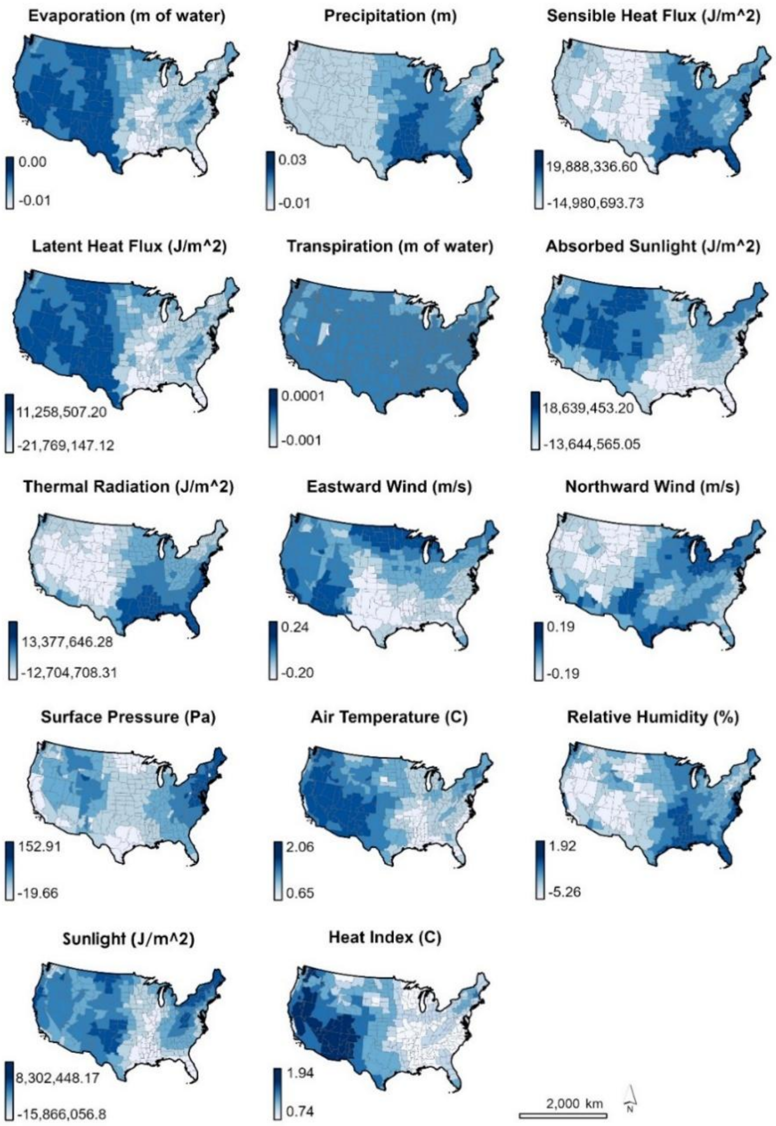

Figure S1: Maps of annual mean anomalies for ERA5-Land climate metrics. Each climate anomaly metric from 1970-1979 base period mean to 2013-2022 recent mean. All metrics were symbolized using Jenks Natural Breaks with 5 classes. All metrics were symbolized using Jenks Natural Breaks with 5 classes.

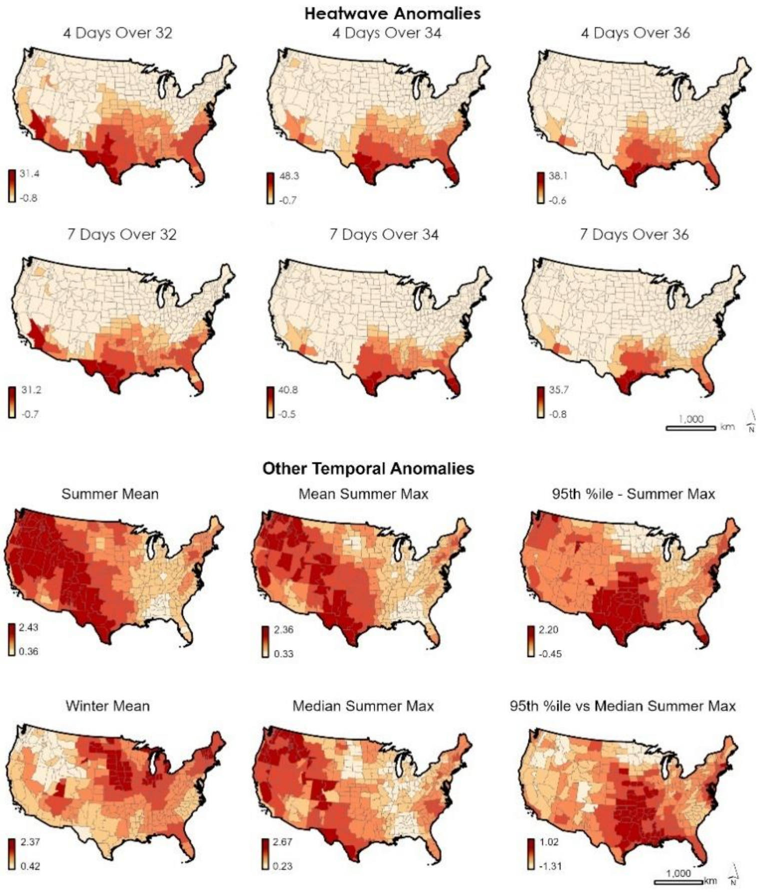

Figure S2: Maps of temporal strata of heat index anomalies. Each climate anomaly metric from 1970-1979 and 2013-2022. All metrics were symbolized using Jenks Natural Breaks with 5 classes.

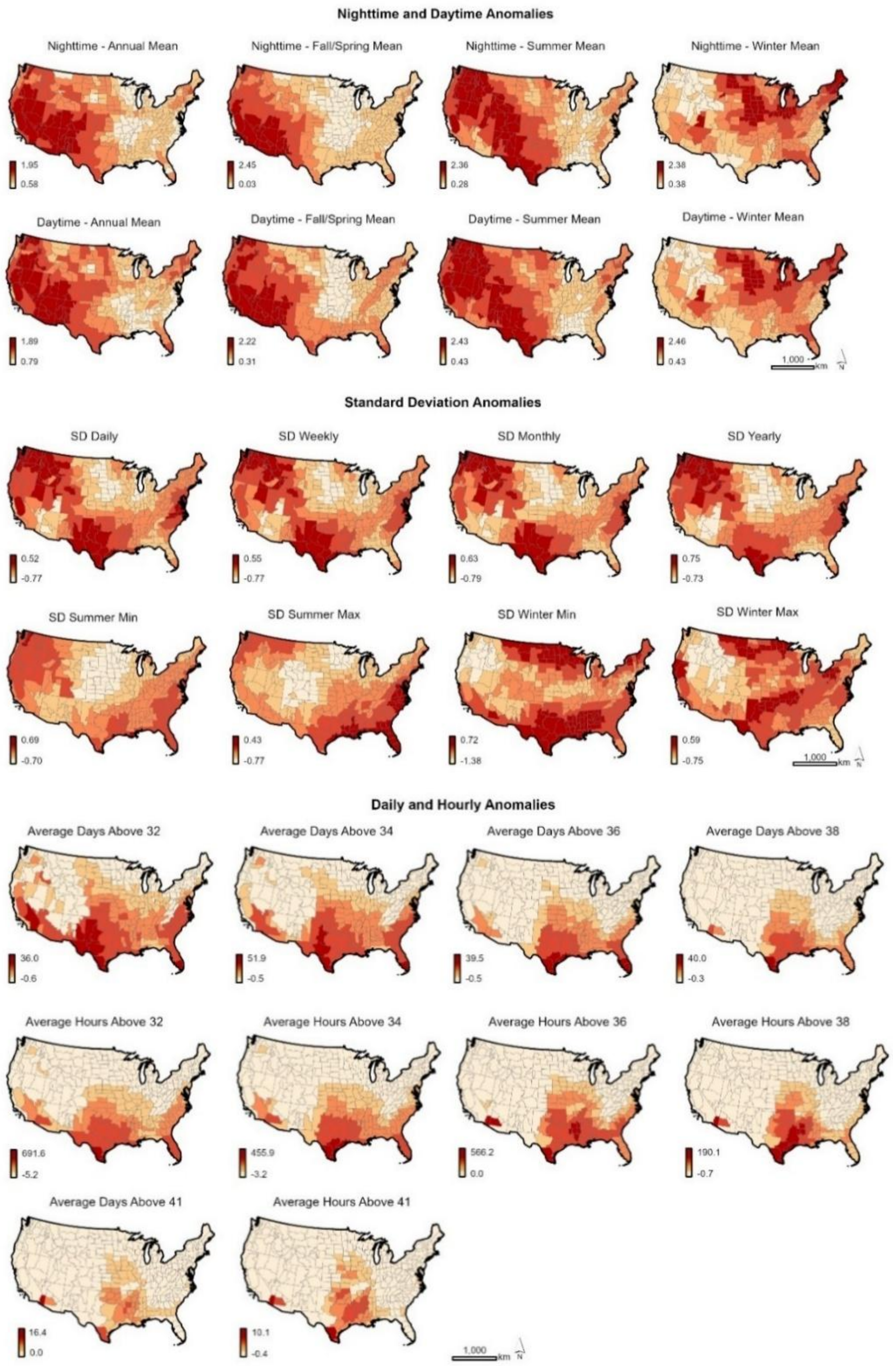

Figure S3: Maps of anomalies for temporal delineations of heat index. Each climate anomaly metric from 1970-1979 and 2013-2022. All metrics were symbolized using Jenks Natural Breaks with 5 classes.

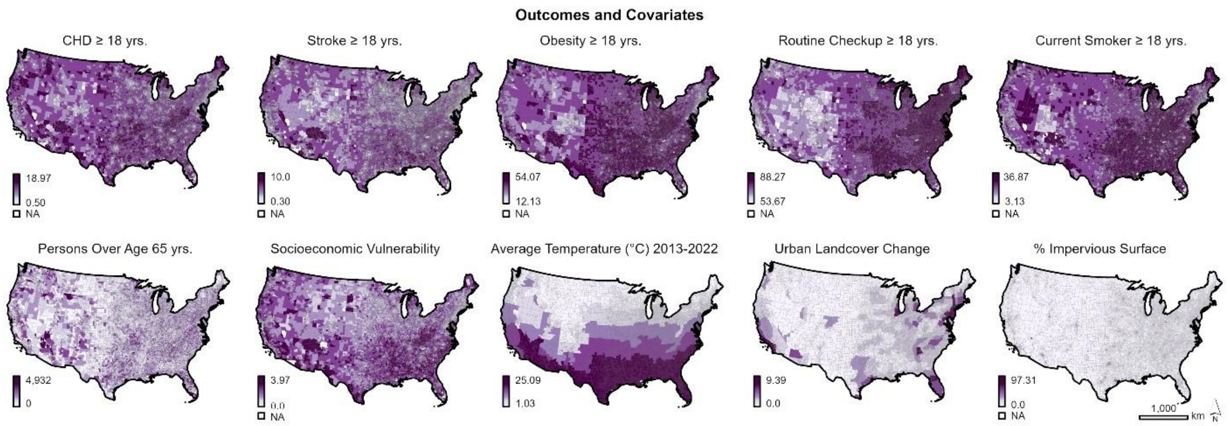

Figure S4: Maps of health outcomes and covariate data. Health outcomes include coronary heart disease and stroke for persons 18 years of age and older. Obesity, routine checkup, and current smoker for person 18 years of age and older as well as persons over age 65, socioeconomic vulnerability, average air temperature (°C) from 2013-2022, urban land cover change, and percentage of impervious surface cover were covariates. All metrics were symbolized using Jenks Natural Breaks with 5 classes. Legends with NA values denote census tracts without data for the metric. Most of the census tracts with missing data were wildlife refuges, Native American reservations, and other areas where data could not be obtained.

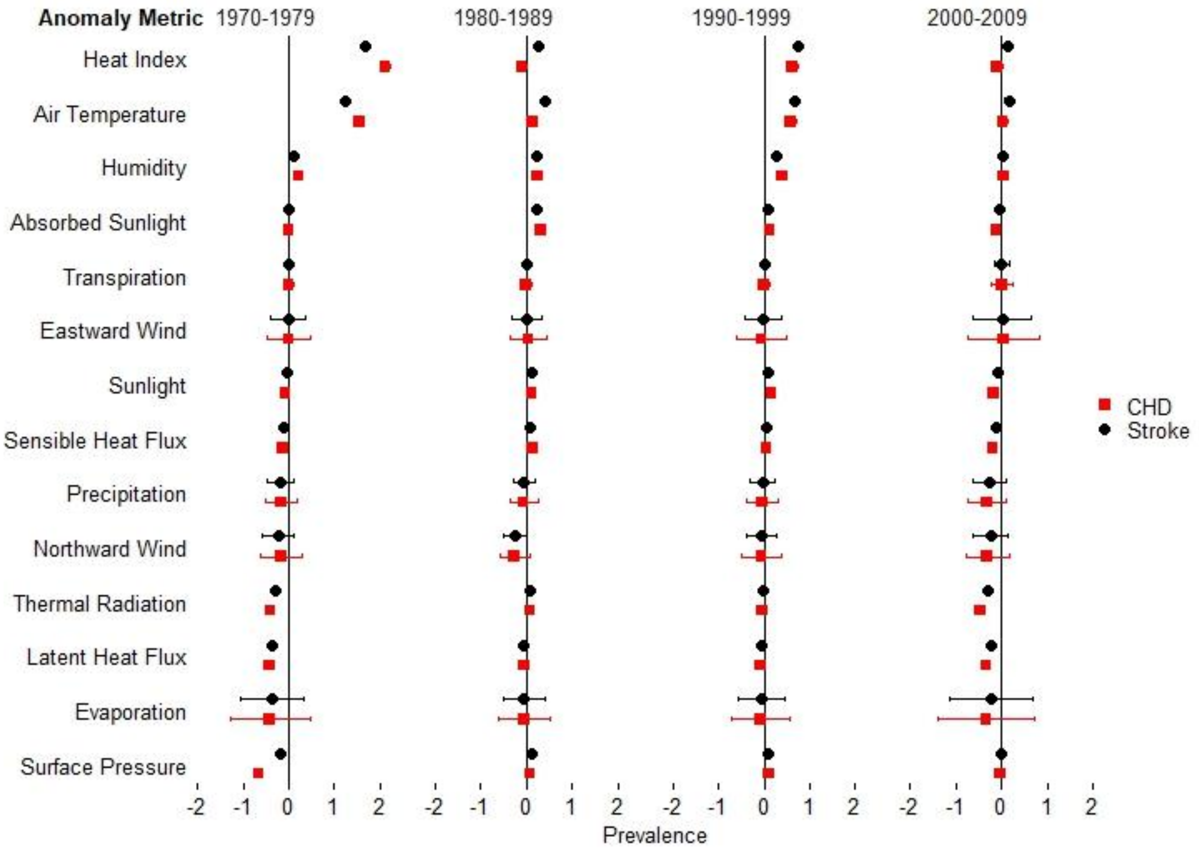

Figure S5: Sensitivity analysis of alternate 10-year baseline time definitions in associations between anomaly metrics and health outcomes. The anomaly effect size was computed as the model coefficient multiplied by the mean anomaly size. The “1970-1979” metrics represent the anomaly time frames used throughout the primary analysis of the difference between the 1970-1979 annual mean and the 2013-2022 annual mean values. As an alternate base time frame, “1980-1989” metrics represent the difference between the 1970-1979 annual mean and the 2013-2022 annual mean values. Similarly, “1990-1999” and “2000-2009” both represent the difference between these respective base time frame annual mean values and 2013-2022 recent annual mean values.

Anomaly Metric	Anomaly Effect Size	CI (95%)
SD Max Winter	-0.01	(-0.1,0.09)
	0	(-0.13,0.12)
SD Min Winter	0.01	(-0.05,0.06)
	0	(-0.07,0.07)
SD Max Summer	0.07	(-0.04,0.18)
	0.15	(0.02,0.28)
SD Min Summer	0.02	(-0.08,0.11)
	0.07	(-0.06,0.2)
SD Yearly	-0.01	(-0.11,0.09)
	-0.01	(-0.13,0.11)
SD Monthly	-0.03	(-0.13,0.07)
	-0.03	(-0.15,0.1)
SD Weekly	-0.04	(-0.14,0.07)
	-0.04	(-0.17,0.09)
SD Daily	-0.04	(-0.14,0.07)
	-0.04	(-0.17,0.09)
7 days ≥ 36	0.06	(0.06,0.07)
	0.09	(0.08,0.1)
4 days ≥ 36	0.09	(0.08,0.09)
	0.12	(0.12,0.13)
7 days ≥ 34	0.08	(0.08,0.09)
	0.15	(0.15,0.16)
4 days ≥ 34	0.08	(0.07,0.08)
	0.19	(0.18,0.19)
7 days ≥ 32	0.04	(0.03,0.04)
	0.1	(0.1,0.11)
4 days ≥ 32	0.06	(0.06,0.07)
	0.17	(0.17,0.18)
95th %ile vs Median Max Summer	-0.01	(-0.05,0.04)
	0.01	(-0.05,0.06)

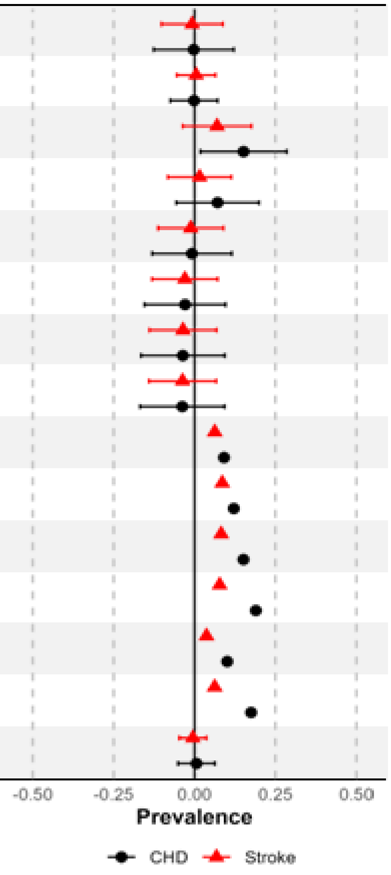

Figure S6: Association of heat wave and variability anomalies with CHD and stroke. Comparison of each temporally delineated climate anomaly metric between a baseline of 1970-1979 and 2013-2022 in separate models. The anomaly effect size was computed as the model coefficient multiplied by the mean anomaly size. All heat index measurements are in units of Celsius. SD=standard deviation. CI = 95% confidence interval.

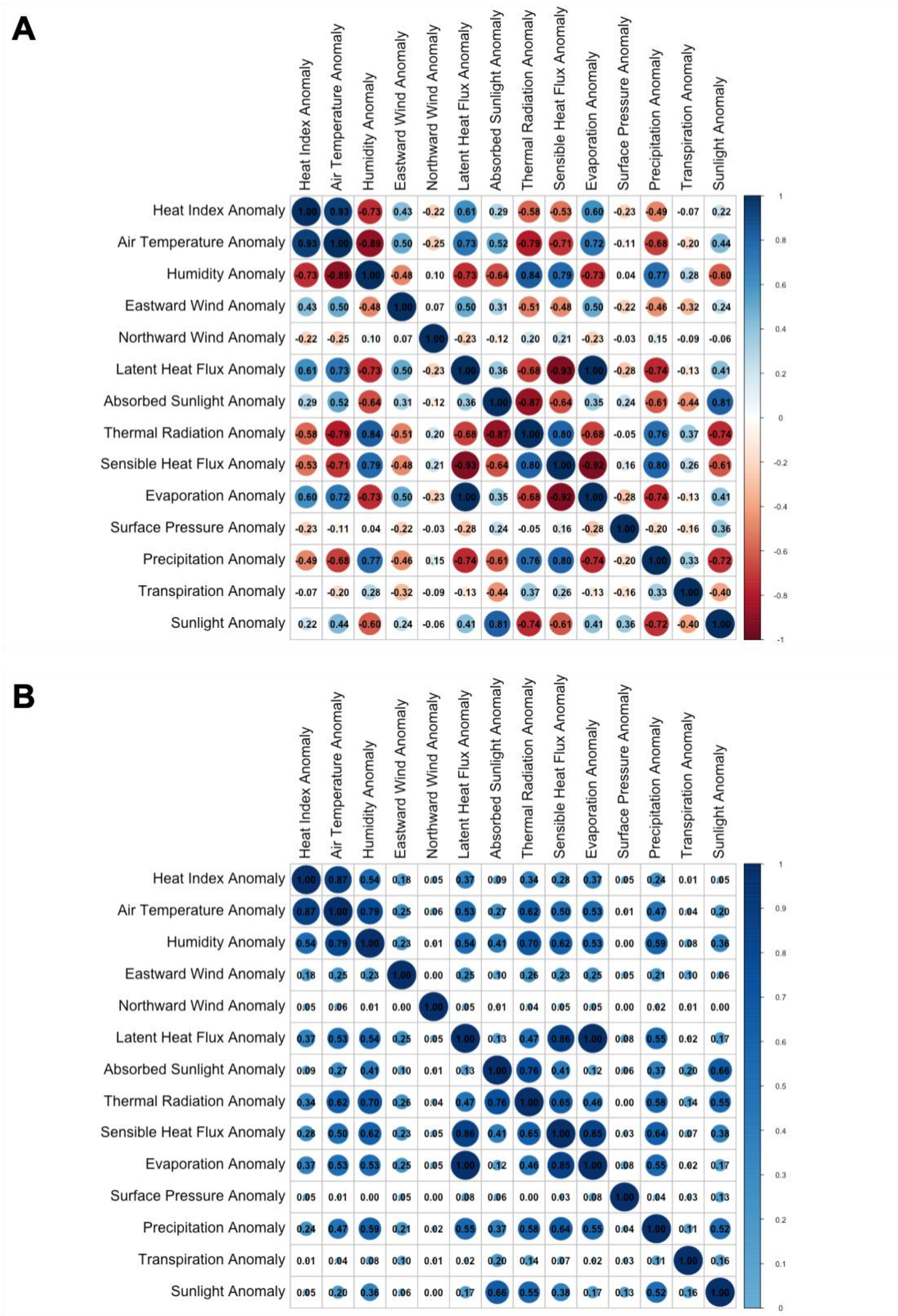

Figure S7a and b: R (a) and R^2 (b) correlation matrices of anomaly metrics considered for mixed-effects linear models. All raw data provided by the EU Copernicus program as part of the ERA5-Land dataset and aggregated at the climate division scale in Google Earth Engine.

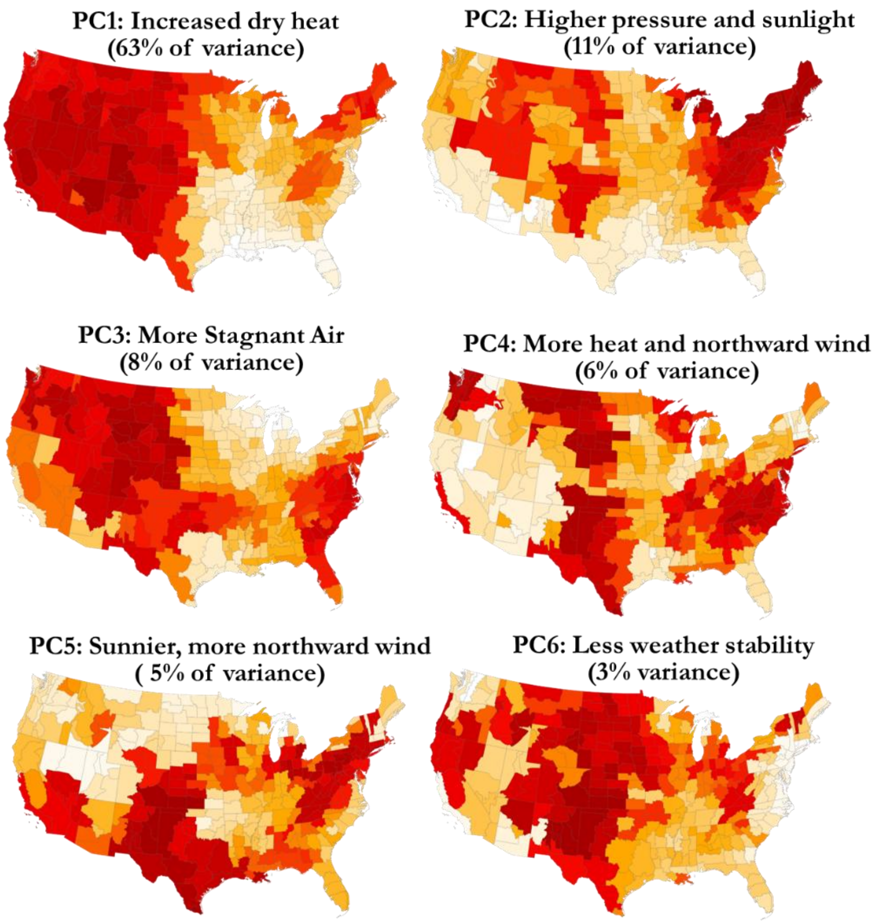

Figure S8: Spatial patterns of co-occurring climate anomalies. Principal components 1-6, variance explained, and scores mapped by climate division symbolized by natural breaks. Darker colors represent higher component loadings. Qualitative descriptions applied based on interpretation of respective principal component factor loadings.

Supplemental Tables

Characteristic	Low	Medium	High
**Tracts (N)**	23,845	23,930	23,510
**Population (N in millions)**	105.31	101.44	113.56
**Sex**			
Male (M)	48.97 (1072.70)	46.99 (1068.40)	54.37 (1316.82)
Female (F)	51.03 (1109.32)	48.98 (1108.63)	55 (1337.2)
**Race and ethnicity**			
White	69.15 (1945.98)<br>[0, 27631] (0.21%)	73.17 (1988.54)<br>[0, 38768] (0.12%)	79.66 (2099.06)<br>[0, 44641] (0.04%)
Black	18.36 (1130.73)<br>[0, 19263] (5.05%)	12.55 (914.80)<br>[0, 13062] (7.27%)	7.97 (668.88)<br>[0, 17568] (10.09%)
Other race	9.51 (733.96)<br>[0, 9103] (5.74%)	7.55 (526.65)<br>[0, 7824] (5.16%)	17.60 (960.09)<br>[0, 21320] (0.94%)
Two or more races	2.99 (136.26)<br>[0, 1606] (6%)	2.71 (131.22)<br>[0, 3555] (5.6%)	4.13 (185.25)<br>[0, 2718] (3.67%)
Hispanic or Latino	9.92 (730.37)<br>[0, 10641] (4.47%)	11.61 (907.25)<br>[0, 14269] (3.44%)	34 (1668.30)<br>[0, 34439] (0.46%)
Not Hispanic or Latino	90.08 (2018.81)<br>[34, 28754] (0%)	84.37 (1975.28)<br>[0, 39061] (0.02%)	75.37 (2247.93)<br>[0, 54518] (0.09%)
**Socioeconomics**			
Median household income	67629.84 (36461.73)<br>[2499, 250001] (0%)	64491.76 (31497.98)<br>[2499, 250001] (0%)	68965.47 (32155.82)<br>[3393, 250001] (0%)
Socioeconomic vulnerability	1.99 (0.98)<br>[0.01, 3.98] (0%)	1.98 (0.97)<br>[0.02, 3.98] (0%)	2.03 (0.98)<br>[0.01, 3.99] (0%)
**Age**			
≥ age 65 yrs.	15.83 (7.02)<br>[0, 90.2] (0.29%)	16.99 (8.01)<br>[0, 87.8] (0.25%)	15.14 (8.74)<br>[0, 100] (0.38%)
**Environment**			
Land cover change	3.33 (2.80)<br>[0.02, 12.75] (0%)	3.47 (3.34)<br>[0.01, 15.13] (0%)	3.69 (3.36)<br>[0, 9.39] (0.08%)
Percent impervious surface	27.23 (23.59)<br>[0.12, 96.36] (0%)	32.73 (26.72)<br>[0.07, 98.36] (0%)	36.77 (25.98)<br>[0.02, 98.46] (0%)
Temperature (°C) 2013-2022	13.98 (3.41)<br>[4.04, 21.15] (0%)	13.24 (4.91)<br>[1.46, 24.37] (0%)	14.90 (5.15)<br>[2.74, 25.08] (0%)
Heat Index 1970-1979 vs 2013-2022	0.92 (0.05)<br>[0.79, 1] (0%)	1.05 (0.04)<br>[1, 1.15] (0%)	1.37 (0.15)<br>[1.15, 1.84] (0%)

Table S1: Demographic and environmental variables by tertiles of the heat index anomaly from 1970-1979 and 2013-2022. The mean and standard deviation in parentheses are shown in the first line for each variable. The range of values is shown in brackets and the percentage of 0 values is shown in parentheses in the second line for each variable.

	Low	Medium	High
Tracts (N)	23,845	23,930	23,510
Population (N in millions)	105.31	101.44	113.56
**Health**			
**Coronary heart disease**	6.40 (2.08)	6.75 (2.08)	5.83 (1.94)
	[3.47, 65.27]	[3.07, 55.87]	[3.8, 63.9]
**Stroke ≥ 18 yrs.**	3.47 (1.38)	3.49 (1.27)	3.06 (1.02)
	[0.3, 14.2]	[0.23, 20.2]	[0.3, 10.93]
Lack of health insurance	14.79 (6.34)	14.62 (7.02)	18.22 (10.41)
	[10.10, 18.40]	[9.57, 18.07]	[10.47, 23.37]
Arthritis	25.90 (6.38)	26.28 (6.29)	22.31 (5.62)
	[3, 52.25]	[2.47, 53.8]	[2.33, 48.97]
Binge drinking	16.96 (3.47)	17.14 (3.08)	17.59 (2.99)
	[2.73, 36.1]	[4.8, 40.8]	[4.13, 35.53]
High blood pressure	34.08 (7.82)	33.44 (7.04)	29.86 (6.18)
	[5.93, 66.97]	[5.27, 71.63]	[5.27, 62.67]
Blood pressure medication	74.22 (6.72)	74.93 (6.00)	69.90 (7.38)
	[11.67, 91.4]	[13.7, 91.3]	[9.23, 92.3]
Cancer	6.52 (1.71)	6.86 (1.84)	6.10 (1.99)
	[0.6, 20.03]	[0.63, 19.6]	[0.53, 20.5]
Asthma ≥ 18 yrs.	9.94 (1.56)	10.15 (1.65)	9.60 (1.30)
	[5.83, 20.5]	[5.7, 20]	[5.3, 18.6]
Cervical cancer screening, F 21–65 yrs.	83.77 (3.97)	83.60 (4.12)	81.77 (4.41)
	[44.7, 92]	[44.13, 92]	[43.5, 91.33]
Routine checkup ≥ 18 yrs.	76.74 (3.84)	77.43 (3.31)	71.02 (4.61)
	[57.67, 91.05]	[58.53, 92.07]	[51.03, 91.63]
Cholesterol screening ≥ 18 yrs.	85.99 (3.98)	86.24 (3.94)	84.13 (4.60)
	[52.33, 98.03]	[52.53, 97.3]	[49.4, 97.23]
Colon screening, 50–75 yrs.	66.30 (5.62)	66.76 (5.85)	62.88 (7.34)
	[33.3, 81.7]	[32.47, 85.13]	[31.17, 82.43]
Chronic obst. pulmonary disease ≥ 18 yrs.	7.34 (2.75)	7.77 (2.82)	6.24 (2.10)
	[1.1, 24.07]	[1.13, 27.17]	[1.13, 20.77]
Clinical preventative services, M ≥ 65 yrs.	37.03 (6.53)	35.27 (6.79)	33.07 (6.67)
	[14.5, 56.67]	[11.97, 57.47]	[11.47, 52.23]
Clinical preventative services, F ≥ 65 yrs.	32.72 (5.91)	30.81 (5.91)	29.91 (5.57)
	[14.93, 52.03]	[8.67, 57.73]	[9.27, 46.27]
Smoking ≥ 18 yrs.	18.63 (6.06)	19.04 (5.94)	15.79 (4.85)
	[3.13, 48.93]	[5.07, 47]	[4.53, 51.93]
Diabetes ≥ 18 yrs.	11.29 (3.92)	11.25 (3.64)	10.47 (3.41)
	[0.95, 36.13]	[0.73, 43.07]	[0.8, 31.03]
Dental care ≥ 18 yrs.	63.11 (10.84)	63.04 (10.80)	61.92 (11.04)
	[18.7, 86.03]	[22.93, 86.33]	[20.53, 84.97]
High cholesterol ≥ 18 yrs.	32.76 (4.87)	32.93 (4.52)	31.52 (4.68)
	[7.5, 53.6]	[6.7, 52.4]	[7.5, 52.43]
Chronic kidney disease ≥ 18 yrs.	3.00 (0.89)	3.07 (0.86)	2.90 (0.70)
	[0.53, 9.87]	[0.53, 13.97]	[0.5, 7.93]
Lack leisure physical activity ≥ 18 yrs.	26.38 (7.77)	27.16 (7.46)	24.18 (7.63)
	[8.73, 60.37]	[9.3, 62.67]	[8.87, 54.8]
Mammogram, women 50–74 yrs.	77.63 (2.31)	77.45 (3.18)	74.63 (3.48)
	[62.7, 87.77]	[55.1, 87.83]	[45.63, 88.1]
Obesity ≥ 18 yrs.	3.38 (0.70)	3.28 (0.67)	3.11 (0.64)
	[1.21, 5.8]	[1.27, 5.71]	[1.2, 5.8]
Poor physical health ≥ 18 yrs., ≥ 14 days	12.45 (3.70)	12.88 (3.69)	12.24 (3.33)
	[3.7, 30.87]	[3.3, 34.63]	[3.83, 32.9]
< 7 hrs. sleep, ≥ 18 yrs.	36.34 (5.73)	36.65 (5.72)	34.27 (4.10)
	[20.07, 58.47]	[21.1, 55.4]	[20.43, 53.23]
All teeth lost ≥ 65 yrs.	15.44 (2.63)	15.65 (1.51)	14.88 (2.04)
	[2.57, 57.13]	[3.03, 54.1]	[2.97, 59.57]
Depression ≥ 18 yrs.	20.81 (3.67)	20.93 (3.63)	19.86 (3.20)
	[8.9, 37.35]	[9.6, 36.3]	[8.4, 35.35]
Poor health ≥ 18 yrs.	17.75 (7.02)	17.95 (6.91)	17.81 (7.14)
	[4.1, 51.5]	[3.5, 56.5]	[4.4, 56.1]

Table S2: Health status and risk variables by tertiles of the heat index anomaly from 1970-1979 and 2013-2022. Values represent tract-level percent of population. The mean and standard deviation in parentheses are shown in the first line for each variable. The range of values is shown in brackets in the second line for each variable.

A. CHD

Model	Effect size per unit change (CI)	T Value	Percent HI contribution	R2	RMSE	AIC	BIC
Unadjusted	-1.14 (-1.21, -1.07)	-31.2	-20.04	0.01	2.05	307,169.8	307,197.3
Demographically adjusted	-1.22 (-1.26, -1.18)	-61.62	-21.5	0.71	1.11	218,562.9	218,608.8
Health adjusted	-0.31 (-0.35, -0.28)	-18.4	-5.53	0.80	0.92	190,904.3	190,959.4
Health behavior adjusted	0.72 (0.68, 0.75)	38.9	12.58	0.85	0.80	171,721.1	171,794.5
Environmentally adjusted	0.71 (0.68, 0.75)	38.94	12.53	0.85	0.80	170,913.8	171,005.6
Land cover change adjusted	0.71 (0.67, 0.75)	38.63	12.46	0.85	0.80	170,906.9	171,007.8
Mixed effects (Census Division)	1.35 (1.24, 1.46)	24.35	23.68	0.18	1.97	301,263.8	301,300.5
Mixed effects (Climate Division)	1.68 (1.44, 1.93)	13.49	29.64	0.89	0.72	158,840.5	158,950.6
Mixed effects (County) with 1970-79 temp. adjustment	-0.43 (-0.68, -0.18)	-3.38	-7.52	0.44	1.51	272,116.2	272,162.1
Mixed effects (County)	1.90 (1.79, 2.02)	33.27	33.44	0.90	0.68	156,044.9	156,155.0
Mixed effects (Census Division + County), non-collinear anomaly metrics	1.05 (0.87, 1.14)	14.28	17.65	0.9	0.68	154403.3	154577.8
Mixed effects (County), non-collinear anomaly metrics	1.46 (1.34, 1.57)	25.48	25.59	0.89	0.68	154,826.5	154,991.8

B. Stroke

Model	Effect size per unit change (CI)	T Value	Percent HI contribution	R2	RMSE	AIC	BIC
Unadjusted	-0.74 (-0.78, -0.7)	-33.46	-24.54	0.02	1.24	234,629.7	234,657.3
Demographically adjusted	-0.87 (-0.9, -0.85)	-66.78	-29.06	0.65	0.73	158,802.5	158,848.4
Health adjusted	-0.26 (-0.28, -0.24)	-23.17	-8.56	0.77	0.60	129,124.2	129,179.2
Health behavior adjusted	0.66 (0.64, 0.69)	53.88	22.07	0.81	0.54	114,181.8	114,255.3
Environmentally adjusted	0.64 (0.62, 0.66)	52.8	21.25	0.82	0.53	111,566.8	111,658.6
Land cover change adjusted	0.67 (0.65, 0.69)	55.55	22.24	0.82	0.52	110,417.0	110,518.0
Mixed effects (Census Division)	0.47 (0.40, 0.54)	13.95	15.64	0.14	1.20	230,072.9	230,109.6
Mixed effects (Climate Division)	2.35 (2.09, 2.62)	17.37	78.12	0.94	0.39	70,224.6	70,334.7
Mixed effects (County) with 1970-79 temp. adjustment	-0.37 (-0.5, -0.23)	-5.31	-6.45	0.32	1.01	212,792.4	212,838.3
Mixed effects (County)	1.5 (1.41, 1.58)	32.58	49.73	0.94	0.34	57,628.2	57,738.4
Mixed effects (Census Division + County), non-collinear anomaly metrics	0.80 (0.71, 0.88)	17.82	26.43	0.96	0.33	54707.7	54882.2
Mixed effects (County), non-collinear anomaly metrics	1.42 (1.35, 1.50)	36.12	47.3	0.94	0.33	55,950.3	56,115.6

Table S3: Sensitivity analysis model fit results from iterations of heat index anomaly linear model development. A, Association between heat index anomaly metrics and prevalence of CHD. B, Associations between heat index anomaly metrics and prevalence of those who have survived a stroke. Unadjusted - linear regression model with no covariates. Demographically adjusted - included socioeconomic status and residents over age 65 as covariates. Health adjusted - included obesity and all demographic covariates. Health behavior adjusted - included previous covariates as well as smoking and checkup prevalence. Environmentally adjusted – previous covariates, percent of impervious surfaces, and the average annual temperature from 2013-2022. Land cover change adjusted – previous covariates and percent impervious surface change 1970-2020. Mixed effects (Census Division) – model mixed effects term at the U.S. Census Division scale. Mixed effects (Climate Division) – Model mixed effects at the U.S. NOAA Climate Division scale. Mixed effects (County) with 1970-79 temp. adjustment – Model mixed effects at the U.S. County scale and also using 1970-1979 mean temperature as a model adjustment (replacing 2013-2022 mean temperature). For county mixed effects terms, the county FIPS code served as the random intercept variable and all covariates were used for the fixed effects in the mixed effects model. Mixed effects (County) – Model mixed effects at the U.S. County scale: this model served as the final model from primary analyses presented in Figures 2 and 3. Mixed effects (Census Division + County), non-collinear anomaly metrics – refers to a model permutation with mixed effects terms for both census division and county. Non-collinear anomaly metrics refer to the mixed effects model with all non-collinear anomaly metrics included. This includes all model parameters as the primary mixed effects model, with the addition of all ERA5-Land climate anomaly variables with anomaly metric $R^2 < 0.4$ and variance inflation factor <10. CI = 95% confidence interval. Mixed effects (County), non-collinear anomaly metrics – reflects the model used for Table 1 with all non-collinear anomaly metrics and a county-level mixed effects term.

A.

	CHD				Stroke			
**Time Period**	**Effect Size per Unit Change**	**CI (95%)**	**T Value**	**Standard Error**	**Effect Size per Unit Change**	**CI (95%)**	**T Value**	**Standard Error**
Heat Index Anomaly 1970-1979 vs 2013-2022	1.46	(1.34, 1.57)	25.48	0.04	1.42	(1.35, 1.50)	36.12	0.06
Heat Index Anomaly 1970-1989 vs 2003-2022	0.78	(0.59, 0.96)	8.25	0.10	0.41	(0.28, 0.54)	6.18	0.07

B.

		CHD			Stroke		
**Anomaly Metric** (scale factor)	**Anomaly Size**	**Effect Size per Unit Change (CI)**	**Anomaly Effect Size (CI)**	**Relative Prevalence**	**Effect Size per Unit Change (CI)**	**Anomaly Effect Size (CI)**	**Relative Prevalence**
**Heat Index Anomaly**	0.83	0.78 (0.59, 0.96)	0.64 (0.46, 0.83)	10.6	0.41 (0.28, 0.54)	0.34 (0.21, 0.47)	10.8
**Eastward Wind Anomaly**	0	3.09 (2.46, 3.71)	0.01 (-0.62, 0.64)	0.13	3.28 (2.77, 3.78)	0.01 (-0.5, 0.51)	0.26
**Northward Wind Anomaly**	0.05	0.56 (0.04, 1.09)	0.03 (-0.5, 0.55)	0.42	-0.68 (-1.09, -0.26)	-0.03 (-0.44, 0.38)	-0.97
**Evaporation Anomaly** (*10)	-0.01	16.28 (15.07, 17.49)	-0.15 (-1.36, 1.06)	-2.46	15.55 (14.68, 16.42)	-0.14 (-1.01, 0.73)	-4.48
**Surface Pressure Anomaly** (*10^{-1})	0.35	-0.21 (-0.29, -0.14)	-0.07 (-0.15, 0)	-1.24	0.08 (0.04, 0.13)	0.03 (-0.01, 0.07)	0.94
**Transpiration Anomaly** (*10^{3})	-1.01	0.02 (0.01, 0.03)	-0.02 (-0.03, -0.01)	-0.36	0.03 (0.02, 0.04)	-0.03 (-0.04, -0.02)	-1.00
**Sunlight Anomaly** (*10^{-6})	1.59	0 (-0.01, 0)	-0.01 (-0.01, 0)	-0.11	0 (-0.01, 0)	0 (-0.01, 0)	-0.14
	**Sum Effect:**	**CHD:**	0.43	6.98	**Stroke:**	0.18	5.41

Table S4: Sensitivity analysis of the association between health metrics and heat index with 20yr climate metric bins. Similar to the 10yr 1970-1979 base and 2013-2022 recent annual mean values used in the primary analyses, these display is of results for the same model but with alternate 20yr base (1970-1989) and 20yr recent (2003-2022) annual mean values. Anomaly effect size represents the mean of the adult population-weighted model coefficient that has been multiplied each metric local anomaly value, resulting in units of percent adult population prevalence. Percent of stroke prevalence represents the anomaly effect size as a percentage of mean outcome prevalence. CI = 95% confidence interval. A, Comparison of the association between the health outcomes of CHD and stroke and the average heat index from 1970-1979 and 2013-2022 and 1970-1989 and 2003-2022. B, Results of models with all noncollinear annual anomaly metrics.

	Effect Size of Anomaly (95% CI)							
	CHD				Stroke			
**Anomaly metric** (scale factor)	**$R^2 < 0.8$**	**$R^2 < 0.6$**	**$R^2 < 0.4$**	**$R^2 < 0.2$**	**$R^2 < 0.8$**	**$R^2 < 0.6$**	**$R^2 < 0.4$**	**$R^2 < 0.2$**
**Heat Index Anomaly**	1.34 (1.14, 1.54)	1.86 (1.7, 2.03)	1.63 (1.52, 1.74)	1.88 (1.77, 1.99)	1.77 (1.63, 1.91)	2.70 (2.59, 2.81)	1.59 (1.51, 1.67)	1.88 (1.81, 1.96)
**Humidity Anomaly**	-0.09 (-0.12, -0.06)	-0.10 (-0.13, -0.07)	-	-	-0.21 (-0.23, -0.19)	-0.24 (-0.25, -0.22)	-	-
**Eastward Wind Anomaly**	0.01 (-0.38, 0.4)	0.01 (-0.38, 0.4)	0.01 (-0.37, 0.4)	0.02 (-0.37, 0.4)	0.01 (-0.28, 0.29)	0.00 (-0.29, 0.3)	0.01 (-0.28, 0.3)	0.01 (-0.31, 0.32)
**Northward Wind Anomaly**	-0.09 (-0.44, 0.27)	-0.09 (-0.44, 0.26)	-0.11 (-0.46, 0.24)	-0.15 (-0.49, 0.19)	-0.15 (-0.41, 0.11)	-0.17 (-0.43, 0.09)	-0.17 (-0.44, 0.09)	-0.23 (-0.51, 0.05)
**Absorbed Sunlight Anomaly**	0.00 (-0.01, 0.01)	0.00 (-0.01, 0)	-	-	0.00 (0, 0.01)	0.00 (0, 0.01)	-	-
**Thermal Radiation Anomaly**	-0.28 (-0.3, -0.27)	-	-	-	-0.35 (-0.36, -0.34)	-0.28 0.00	-	-
**Evaporation Anomaly**	-0.14 (-1.52, 1.24)	-0.22 (-1.38, 0.94)	-0.17 (-1.2, 0.85)	-	-0.21 (-1.14, 0.71)	-0.41 (-1.08, 0.53)	-0.29 (-1, 0.43)	-
**Surface Pressure Anomaly**	-0.74 (-0.75, -0.73)	-0.72 (-0.73, -0.72)	-0.76 (-0.77, -0.75)	-0.86 (-0.87, -0.85)	-0.38 (-0.38, -0.37)	-0.06 (-0.41, -0.4)	-0.34 (-0.34, -0.33)	-0.49 (-0.5, -0.49)
**Precipitation Anomaly**	0.01 (-0.48, 0.5)	0.03 (-0.41, 0.48)	-	-	-0.13 (-0.47, 0.22)	0.00 (-0.38, 0.26)	-	-
**Transpiration Anomaly**	-0.01 (-0.11, 0.1)	0.00 (-0.1, 0.1)	0.01 (-0.09, 0.1)	0.00 (-0.1, 0.1)	-0.01 (-0.07, 0.05)	0.00 (-0.06, 0.07)	0.00 (-0.06, 0.06)	0.00 (-0.07, 0.06)
**Sunlight Anomaly**	0.03 (0.02, 0.04)	-	-0.02 (-0.02, -0.01)	-0.07 (-0.07, -0.06)	0.15 (0.14, 0.15)	-	0.08 (0.08, 0.09)	0.00 (-0.01, 0)
**Sum Effects (Relative Prevalence):**	0.04 (0.67)	0.77 (12.71)	0.59 (9.84)	0.82 (13.68)	0.49 (15.73)	1.54 (49.38)	0.88 (27.88)	1.17 (36.83)

Table S5. Sensitivity analysis of comprehensive model with permutations of R^2 exclusion criteria. Anomaly effect size represents the population-weighted model coefficient of each metric multiplied by the metric anomaly size for persons aged 18 and older in respective units, resulting in units of percent population prevalence. Relative prevalence represents the anomaly effect size as a percentage of the mean outcome prevalence. CI = 95% confidence interval. Variables excluded by respective cutoff thresholds are represented as missing in the table by " - " symbology.

	Anomaly Effect Size (95% CI)							
	CHD				Stroke			
	All Counties	Bottom 50th Percentile Counties			All Counties	Bottom 50th Percentile Counties		
**Anomaly Metric**	**(Figure 2)**	**IM**	**OM**	**CM**	**(Figure 2)**	**IM**	**OM**	**CM**
**Heat Index**	2.12<br>(2.00, 2.23)	1.49<br>(1.34, 1.64)	1.55<br>(1.40, 1.71)	1.41<br>(1.25, 1.58)	1.66<br>(1.58, 1.74)	0.89<br>(0.78, 1.00)	0.82<br>(0.71, 0.93)	0.74<br>(0.62, 0.85)
**Air Temperature**	1.54<br>(1.45, 1.64)	1.05<br>(0.92, 1.18)	1.09<br>(0.95, 1.22)	0.98<br>(0.84, 1.12)	1.26<br>(1.19, 1.33)	0.67<br>(0.58, 0.77)	0.62<br>(0.53, 0.72)	0.55<br>(0.45, 0.65)
**Humidity**	0.22<br>(0.20, 0.24)	0.19<br>(0.17, 0.21)	0.17<br>(0.15, 0.19)	0.18<br>(0.15, 0.20)	0.12<br>(0.10, 0.13)	0.07<br>(0.05, 0.08)	0.06<br>(0.05, 0.08)	0.05<br>(0.03, 0.07)
**Eastward Wind**	-0.01<br>(-0.48, 0.47)	0.02<br>(-0.61, 0.64)	0.03<br>(-0.64, 0.69)	0.03<br>(-0.65, 0.72)	-0.01<br>(-0.38, 0.36)	0.02<br>(-0.52, 0.55)	0.02<br>(-0.51, 0.56)	0.03<br>(-0.54, 0.60)
**Northward Wind**	-0.16<br>(-0.61, 0.28)	-0.23<br>(-0.84, 0.38)	-0.30<br>(-0.91, 0.30)	-0.30<br>(-0.95, 0.35)	-0.22<br>(-0.57, 0.13)	-0.19<br>(-0.71, 0.33)	-0.24<br>(-0.74, 0.25)	-0.20<br>(-0.75, 0.34)
**Latent Heat Flux**	-0.41<br>(-0.41, -0.41)	-0.33<br>(-0.33, -0.32)	-0.31<br>(-0.31, -0.30)	-0.30<br>(-0.31, -0.30)	-0.37<br>(-0.38, -0.37)	-0.28<br>(-0.29, -0.28)	-0.27<br>(-0.27, -0.26)	-0.24<br>(-0.25, -0.24)
**Surface-Absorbed Sunlight**	0.01<br>(0.00, 0.01)	0.04<br>(0.04, 0.05)	0.04<br>(0.04, 0.05)	0.05<br>(0.05, 0.06)	0.00<br>(0.00, 0.01)	0.02<br>(0.01, 0.02)	0.01<br>(0.01, 0.02)	0.02<br>(0.02, 0.03)
**Thermal Radiation**	-0.40<br>(-0.41, -0.39)	-0.27<br>(-0.28, -0.26)	-0.26<br>(-0.27, -0.25)	-0.24<br>(-0.25, -0.23)	-0.28<br>(-0.29, -0.28)	-0.18<br>(-0.18, -0.17)	-0.15<br>(-0.15, -0.14)	-0.15<br>(-0.16, -0.14)
**Sensible Heat Flux**	-0.12<br>(-0.13, -0.12)	-0.07<br>(-0.07, -0.06)	-0.06<br>(-0.06, -0.05)	-0.05<br>(-0.05, -0.04)	-0.11<br>(-0.11, -0.10)	-0.05<br>(-0.05, -0.04)	-0.04<br>(-0.05, -0.04)	-0.03<br>(-0.04, -0.03)
**Evaporation**	-0.41<br>(-1.29, 0.46)	-0.33<br>(-1.50, 0.84)	-0.31<br>(-1.61, 0.99)	-0.31<br>(-1.67, 1.06)	-0.38<br>(-1.07, 0.32)	-0.29<br>(-1.32, 0.75)	-0.27<br>(-1.32, 0.79)	-0.25<br>(-1.42, 0.93)
**Surface Pressure**	-0.65<br>(-0.66, -0.65)	-0.54<br>(-0.55, -0.53)	-0.55<br>(-0.56, -0.54)	-0.53<br>(-0.54, -0.52)	-0.19<br>(-0.20, -0.19)	-0.25<br>(-0.26, -0.25)	-0.19<br>(-0.20, -0.18)	-0.23<br>(-0.24, -0.22)
**Precipitation**	-0.16<br>(-0.51, 0.19)	-0.07<br>(-0.51, 0.37)	-0.06<br>(-0.54, 0.42)	-0.05<br>(-0.55, 0.44)	-0.17<br>(-0.46, 0.12)	-0.07<br>(-0.49, 0.35)	-0.08<br>(-0.50, 0.35)	-0.06<br>(-0.52, 0.41)
**Transpiration**	-0.01<br>(-0.11, 0.10)	-0.02<br>(-0.17, 0.13)	-0.02<br>(-0.16, 0.13)	-0.02<br>(-0.17, 0.13)	0.01<br>(-0.05, 0.06)	-0.01<br>(-0.10, 0.08)	0.00<br>(-0.09, 0.08)	0.00<br>(-0.09, 0.08)
**Sunlight**	-0.07<br>(-0.08, -0.07)	-0.03<br>(-0.04, -0.02)	-0.01<br>(-0.02, -0.00)	-0.01<br>(-0.02, -0.01)	-0.04<br>(-0.04, -0.03)	0.00<br>(-0.00, 0.01)	0.01<br>(0.00, 0.02)	0.01<br>(0.00, 0.02)

Table S6. Sensitivity analysis of associations between anomalies and outcomes for areas with low in migration (IM), out migration (OM), and combined migration (CM). The anomaly effect size was computed as the model coefficient multiplied by the mean anomaly size. Results reflect the same statistical tests used to generate Figure 2, which is included above for comparison, but among only tracts within counties of the lowest 50th percentile for each type of population migration.

	Anomaly Effect Size (95% CI)							
	CHD				Stroke			
	All Counties	Top 50th Percentile Counties			All Counties	Top 50th Percentile Counties		
**Anomaly Metric**	**(Figure 2)**	**IM**	**OM**	**CM**	**(Figure 2)**	**IM**	**OM**	**CM**
**Heat Index**	2.12<br>(2.00, 2.23)	3.14<br>(2.96, 3.32)	2.74<br>(2.57, 2.92)	3.13<br>(2.94, 3.32)	1.66<br>(1.58, 1.74)	2.67<br>(2.56, 2.79)	2.60<br>(2.48, 2.71)	2.77<br>(2.65, 2.89)
**Air Temperature**	1.54<br>(1.45, 1.64)	2.27<br>(2.12, 2.43)	2.02<br>(1.87, 2.16)	2.27<br>(2.11, 2.44)	1.26<br>(1.19, 1.33)	1.94<br>(1.84, 2.04)	1.93<br>(1.83, 2.02)	2.02<br>(1.91, 2.13)
**Humidity**	0.22<br>(0.20, 0.24)	0.20<br>(0.17, 0.23)	0.19<br>(0.16, 0.22)	0.20<br>(0.17, 0.23)	0.12<br>(0.10, 0.13)	0.14<br>(0.12, 0.16)	0.15<br>(0.13, 0.17)	0.15<br>(0.12, 0.17)
**Eastward Wind**	-0.01<br>(-0.48, 0.47)	-0.01<br>(-0.79, 0.77)	0.01<br>(-0.71, 0.73)	0.00<br>(-0.83, 0.82)	-0.01<br>(-0.38, 0.36)	-0.05<br>(-0.59, 0.48)	-0.04<br>(-0.59, 0.50)	-0.06<br>(-0.65, 0.53)
**Northward Wind**	-0.16<br>(-0.61, 0.28)	-0.10<br>(-0.87, 0.67)	-0.04<br>(-0.77, 0.68)	-0.07<br>(-0.89, 0.75)	-0.22<br>(-0.57, 0.13)	-0.15<br>(-0.67, 0.37)	-0.13<br>(-0.66, 0.40)	-0.13<br>(-0.69, 0.43)
**Latent Heat Flux**	-0.41<br>(-0.41, -0.41)	-0.58<br>(-0.58, -0.57)	-0.55<br>(-0.56, -0.54)	-0.60<br>(-0.61, -0.60)	-0.37<br>(-0.38, -0.37)	-0.47<br>(-0.47, -0.47)	-0.47<br>(-0.48, -0.47)	-0.50<br>(-0.51, -0.50)
**Surface-Absorbed Sunlight**	0.01<br>(0.00, 0.01)	-0.07<br>(-0.08, -0.06)	-0.08<br>(-0.09, -0.07)	-0.08<br>(-0.09, -0.07)	0.00<br>(0.00, 0.01)	-0.04<br>(-0.05, -0.04)	-0.06<br>(-0.06, -0.05)	-0.06<br>(-0.06, -0.05)
**Thermal Radiation**	-0.40<br>(-0.41, -0.39)	-0.62<br>(-0.63, -0.61)	-0.61<br>(-0.62, -0.61)	-0.66<br>(-0.67, -0.65)	-0.28<br>(-0.29, -0.28)	-0.43<br>(-0.44, -0.42)	-0.49<br>(-0.50, -0.49)	-0.50<br>(-0.51, -0.50)
**Sensible Heat Flux**	-0.12<br>(-0.13, -0.12)	-0.23<br>(-0.24, -0.23)	-0.22<br>(-0.23, -0.22)	-0.25<br>(-0.25, -0.24)	-0.11<br>(-0.11, -0.10)	-0.19<br>(-0.20, -0.19)	-0.19<br>(-0.20, -0.19)	-0.21<br>(-0.21, -0.20)
**Evaporation**	-0.41<br>(-1.29, 0.46)	-0.58<br>(-2.05, 0.89)	-0.55<br>(-1.88, 0.77)	-0.61<br>(-2.18, 0.97)	-0.38<br>(-1.07, 0.32)	-0.47<br>(-1.45, 0.50)	-0.47<br>(-1.43, 0.48)	-0.50<br>(-1.58, 0.58)
**Surface Pressure**	-0.65<br>(-0.66, -0.65)	-0.84<br>(-0.86, -0.83)	-0.93<br>(-0.95, -0.92)	-1.00<br>(-1.01, -0.98)	-0.19<br>(-0.20, -0.19)	-0.18<br>(-0.19, -0.17)	-0.24<br>(-0.25, -0.23)	-0.25<br>(-0.26, -0.24)
**Precipitation**	-0.16<br>(-0.51, 0.19)	-0.35<br>(-0.94, 0.23)	-0.31<br>(-0.83, 0.21)	-0.37<br>(-0.99, 0.25)	-0.17<br>(-0.46, 0.12)	-0.33<br>(-0.71, 0.06)	-0.30<br>(-0.68, 0.08)	-0.34<br>(-0.76, 0.08)
**Transpiration**	-0.01<br>(-0.11, 0.10)	0.01<br>(-0.14, 0.15)	0.00<br>(-0.15, 0.16)	0.01<br>(-0.14, 0.16)	0.01<br>(-0.05, 0.06)	0.01<br>(-0.06, 0.09)	0.01<br>(-0.07, 0.10)	0.01<br>(-0.07, 0.09)
**Sunlight**	-0.07<br>(-0.08, -0.07)	-0.15<br>(-0.16, -0.14)	-0.19<br>(-0.20, -0.18)	-0.19<br>(-0.20, -0.18)	-0.04<br>(-0.04, -0.03)	-0.10<br>(-0.11, -0.09)	-0.13<br>(-0.14, -0.13)	-0.14<br>(-0.14, -0.13)

Table S7. Sensitivity analysis of associations between anomalies and outcomes for areas with high in migration (IM), out migration (OM), and combined migration (CM). The anomaly effect size was computed as the model coefficient multiplied by the mean anomaly size. Results reflect the same statistical tests used to generate Figure 2, which is included above for comparison, but among only tracts within counties of the highest 50th percentile for each type of population migration.

A.

Anomaly Effect Size (95% CI) CHD							
	All Tracts (Figure 2)	Socioeconomic Vulnerability		Mean Annual Temperature (°C) 2013-2022		RUCA	
**Anomaly Metric**		**Low**	**High**	**Low**	**High**	**Less Dense**	**Urban Core**
**Heat Index**	2.12 (2.00, 2.23)	2.23 (2.07, 2.38)	2.01 (1.89, 2.13)	1.94 (1.77, 2.11)	2.36 (2.20, 2.52)	3.70 (3.54, 3.86)	1.70 (1.54, 1.87)
**Air Temperature**	1.54 (1.45, 1.64)	1.60 (1.47, 1.74)	1.49 (1.39, 1.60)	1.55 (1.40, 1.69)	1.67 (1.53, 1.80)	2.84 (2.70, 2.98)	1.26 (1.11, 1.40)
**Humidity**	0.22 (0.20, 0.24)	0.20 (0.17, 0.22)	0.21 (0.19, 0.23)	0.30 (0.27, 0.33)	0.20 (0.18, 0.23)	0.43 (0.40, 0.46)	0.15 (0.12, 0.18)
**Eastward Wind**	-0.01 (-0.48, 0.47)	-0.01 (-0.66, 0.65)	-0.01 (-0.48, 0.46)	0.01 (-0.73, 0.74)	0.00 (-0.69, 0.70)	-0.01 (-0.68, 0.67)	-0.02 (-0.73, 0.69)
**Northward Wind**	-0.16 (-0.61, 0.28)	-0.27 (-0.88, 0.34)	-0.13 (-0.58, 0.32)	-0.50 (-0.99, -0.00)	0.26 (-0.57, 1.10)	-0.30 (-0.94, 0.34)	-0.11 (-0.84, 0.62)
**Latent Heat Flux**	-0.41 (-0.41, -0.41)	-0.53 (-0.54, -0.53)	-0.37 (-0.37, -0.37)	-0.64 (-0.64, -0.63)	-0.31 (-0.32, -0.31)	-0.79 (-0.80, -0.79)	-0.30 (-0.30, -0.29)
**Surface-Absorbed Sunlight**	0.01 (0.00, 0.01)	0.04 (0.04, 0.05)	-0.02 (-0.02, -0.01)	0.22 (0.21, 0.23)	-0.20 (-0.20, -0.19)	0.12 (0.11, 0.13)	-0.01 (-0.02, -0.01)
**Thermal Radiation**	-0.40 (-0.41, -0.39)	-0.43 (-0.43, -0.42)	-0.41 (-0.41, -0.40)	-0.26 (-0.26, -0.25)	-0.65 (-0.66, -0.64)	-0.65 (-0.66, -0.64)	-0.33 (-0.34, -0.33)
**Sensible Heat Flux**	-0.12 (-0.13, -0.12)	-0.15 (-0.15, -0.14)	-0.11 (-0.12, -0.11)	-0.13 (-0.13, -0.12)	-0.13 (-0.13, -0.12)	-0.20 (-0.20, -0.19)	-0.10 (-0.11, -0.09)
**Evaporation**	-0.41 (-1.29, 0.46)	-0.54 (-1.87, 0.80)	-0.37 (-1.31, 0.57)	-0.64 (-2.00, 0.72)	-0.31 (-1.54, 0.91)	-0.80 (-2.03, 0.44)	-0.30 (-1.77, 1.17)
**Surface Pressure**	-0.65 (-0.66, -0.65)	-0.57 (-0.58, -0.56)	-0.66 (-0.67, -0.65)	-0.61 (-0.62, -0.59)	-0.90 (-0.91, -0.88)	-0.87 (-0.88, -0.85)	-0.43 (-0.44, -0.42)
**Precipitation**	-0.16 (-0.51, 0.19)	-0.13 (-0.66, 0.39)	-0.16 (-0.50, 0.17)	-0.07 (-0.63, 0.50)	-0.31 (-0.78, 0.17)	-0.37 (-0.88, 0.14)	-0.12 (-0.66, 0.41)
**Transpiration**	-0.01 (-0.11, 0.10)	0.01 (-0.08, 0.11)	0.00 (-0.17, 0.17)	0.01 (-0.10, 0.12)	0.01 (-0.68, 0.71)	-0.04 (-0.26, 0.18)	0.00 (-0.12, 0.13)
**Sunlight**	-0.07 (-0.08, -0.07)	-0.06 (-0.07, -0.05)	-0.07 (-0.07, -0.06)	-0.01 (-0.01, 0.00)	-0.27 (-0.27, -0.26)	-0.09 (-0.10, -0.08)	-0.08 (-0.09, -0.07)

B.

Anomaly Effect Size (95% CI) Stroke							
	All Tracts (Figure 2)	Socioeconomic Vulnerability		Mean Annual Temperature (°C) 2013-2022		RUCA	
**Anomaly Metric**		**Low**	**High**	**Low**	**High**	**Less Dense**	**Urban Core**
**Heat Index**	1.66 (1.58, 1.74)	1.06 (0.99, 1.14)	2.38 (2.28, 2.49)	2.13 (1.99, 2.26)	1.51 (1.41, 1.61)	2.33 (2.22, 2.43)	0.95 (0.82, 1.09)
**Air Temperature**	1.26 (1.19, 1.33)	0.80 (0.74, 0.86)	1.81 (1.72, 1.90)	1.79 (1.68, 1.90)	1.23 (1.15, 1.31)	1.85 (1.76, 1.94)	0.72 (0.61, 0.83)
**Humidity**	0.12 (0.10, 0.13)	0.08 (0.07, 0.09)	0.19 (0.17, 0.20)	0.19 (0.17, 0.21)	0.14 (0.12, 0.16)	0.23 (0.21, 0.24)	0.06 (0.04, 0.08)
**Eastward Wind**	-0.01 (-0.38, 0.36)	-0.01 (-0.33, 0.31)	-0.02 (-0.54, 0.50)	0.01 (-0.62, 0.63)	-0.04 (-0.54, 0.46)	-0.01 (-0.45, 0.42)	-0.02 (-0.64, 0.59)
**Northward Wind**	-0.22 (-0.57, 0.13)	-0.17 (-0.47, 0.12)	-0.27 (-0.76, 0.21)	-0.55 (-0.95, -0.15)	0.11 (-0.50, 0.72)	-0.27 (-0.71, 0.17)	-0.15 (-0.76, 0.46)
**Latent Heat Flux**	-0.37 (-0.38, -0.37)	-0.28 (-0.29, -0.28)	-0.48 (-0.49, -0.48)	-0.73 (-0.74, -0.73)	-0.30 (-0.30, -0.30)	-0.52 (-0.53, -0.52)	-0.28 (-0.28, -0.27)
**Surface-Absorbed Sunlight**	0.00 (0.00, 0.01)	0.02 (0.01, 0.02)	-0.01 (-0.02, -0.01)	0.16 (0.15, 0.17)	-0.09 (-0.10, -0.09)	0.07 (0.06, 0.07)	0.00 (-0.01, 0.00)
**Thermal Radiation**	-0.28 (-0.29, -0.28)	-0.20 (-0.21, -0.20)	-0.41 (-0.41, -0.40)	-0.27 (-0.28, -0.26)	-0.40 (-0.41, -0.40)	-0.41 (-0.42, -0.41)	-0.17 (-0.17, -0.16)
**Sensible Heat Flux**	-0.11 (-0.11, -0.10)	-0.07 (-0.08, -0.07)	-0.15 (-0.15, -0.14)	-0.12 (-0.13, -0.12)	-0.12 (-0.13, -0.12)	-0.13 (-0.13, -0.13)	-0.08 (-0.09, -0.08)
**Evaporation**	-0.38 (-1.07, 0.32)	-0.29 (-0.94, 0.37)	-0.48 (-1.37, 0.40)	-0.73 (-1.79, 0.32)	-0.30 (-1.10, 0.50)	-0.53 (-1.35, 0.30)	-0.28 (-1.54, 0.98)
**Surface Pressure**	-0.19 (-0.20, -0.19)	-0.12 (-0.13, -0.12)	-0.48 (-0.49, -0.47)	-0.16 (-0.17, -0.15)	-0.53 (-0.54, -0.52)	-0.20 (-0.20, -0.19)	-0.23 (-0.24, -0.22)
**Precipitation**	-0.17 (-0.46, 0.12)	-0.08 (-0.34, 0.17)	-0.25 (-0.62, 0.11)	-0.08 (-0.55, 0.39)	-0.34 (-0.66, -0.01)	-0.27 (-0.63, 0.08)	-0.11 (-0.57, 0.36)
**Transpiration**	0.01 (-0.05, 0.06)	0.01 (-0.03, 0.05)	-0.01 (-0.14, 0.13)	0.02 (-0.05, 0.09)	0.02 (-0.48, 0.52)	-0.01 (-0.12, 0.11)	0.00 (-0.07, 0.08)
**Sunlight**	-0.04 (-0.04, -0.03)	-0.03 (-0.03, -0.02)	-0.03 (-0.03, -0.02)	-0.01 (-0.02, -0.00)	-0.16 (-0.16, -0.15)	-0.04 (-0.04, -0.03)	-0.01 (-0.02, -0.00)

Table S8a and S8b. Stratification of associations between anomalies by demographic variables for CHD (a) and stroke (b). The anomaly effect size was computed as the model coefficient multiplied by the mean anomaly size. Results reflect the same statistical tests used to generate Figure 2, which is included above for comparison, but tracts are stratified to low and high socioeconomic vulnerability and mean annual temperature (°C) values from 2013-2022. Tracts were also stratified by their RUCA classification code to urban core (representing core metropolitan areas) and less dense (representing all other RUCA codes).

	PC1	PC2	PC3	PC4	PC5	PC6
PCA Proportion	0.6279	0.1072	0.078	0.0594	0.0539	0.0328
Heat Index	0.2804	-0.26	-0.128	0.4735	-0.265	0.0111
Temperature	0.3093	-0.163	-0.133	0.3758	-0.245	0.0096
Humidity	-0.321	-0.012	0.1344	-0.235	0.05	-0.221
Eastward wind	0.1596	-0.169	-0.662	-0.513	-0.205	-0.406
Northward wind	-0.177	0.0819	-0.517	0.4387	0.6472	-0.167
Latent Heat Flux	0.3107	-0.146	0.2935	0.002	0.215	-0.356
Absorbed Sunlight	0.3036	0.1913	-0.15	-0.207	0.0383	0.473
Thermal Radiarion	-0.337	-0.029	0.085	0.0449	0.0365	-0.203
Sensible heat flux	-0.327	-0.008	-0.176	0.142	-0.248	0.0241
Evaporation	0.3104	-0.147	0.2928	-1E-03	0.2167	-0.359
Surface Pressure	0.0019	0.7457	0.0444	0.2118	-0.386	-0.38
Precipitation	-0.306	-0.221	0.0423	0.0421	-0.064	0.2365
Sunlight	0.2596	0.4305	-0.071	-0.11	0.3088	0.1906

Table S9: Principal components analysis loadings. Component loadings for each climate anomaly metric in PCA for the first 8 principal components.

Component	Eigenvalue	Proportion of variance	CHD Estimate	CHD Estimate*variance	Stroke Estimate	Stroke Estimate*variance
PC1	8.162352	0.627870	0.172595	0.108367	0.108367	0.068041
PC2	1.393388	0.107180	-0.254241	-0.027250	-0.027250	-0.002921
PC3	1.014282	0.078020	0.126324	0.009856	0.009856	0.000769
PC4	0.771915	0.059380	0.121827	0.007234	0.007234	0.000430
PC5	0.700766	0.053910	0.126935	0.006843	0.006843	0.000369
PC6	0.425828	0.032760	0.146741	0.004807	0.004807	0.000157
PC7	0.224980	0.017310	-0.109126	-0.001889	-0.001889	-0.000033
PC8	0.135483	0.010420	-0.087745	-0.000914	-0.000914	-0.000010

Table S10: Random intercepts model of principal components scores sensitivity analysis results. Random intercepts regression model results for each principal component where PCA component scores replaced climate anomaly metrics in the model. For CHD and Stroke outcomes in separate models. Effect size reflects model coefficient and not effect of anomaly. PCA results of variance explained and eigenvalues for each component are included for context. For interpretation of relative overall importance, “Outcome Estimate*variance” column represents the effect size multiplied by the variance explained by each principal component.

Variable	Scale (10-yr model)	Unit	Source dataset	Dataset variable name	Year(s)	Spatial resolution
Coronary heart disease prevalence	N/A	Percent of population	CDC PLACES	CHD_CrudePrev	2020-2022	Census tracts (2010)
Stroke prevalence	N/A	Percent of population	CDC PLACES	STROKE_CrudePrev	2020-2022	Census tracts (2010)
Current smoker	N/A	Percent of population	CDC PLACES	CSMOKING_CrudePrev	2020-2022	Census tracts (2010)
Routine checkup	N/A	Percent of population	CDC PLACES	CHECKING_CrudePrev	2020-2022	Census tracts (2010)
Obesity	$*10^{-1}$	Percent of population	CDC PLACES	OBESITY_CrudePrev	2020-2022	Census tracts (2010)
Social vulnerability	N/A	Index value	CDC Social Vulnerability Index	SPL_THEME1	2018	Census tracts (2010)
Persons over age 65	N/A	Percent of population	CDC Social Vulnerability Index	EP_AGE65	2018	Census tracts (2010)
Percent impervious surfaces	N/A	Percent impervious surface	ESRI Living Atlas of the World, Climate Resilience	PCT_ImperviousSurfaces	2024	Census tracts (2010)

Table S11: Health outcomes and covariates used in the analysis.

Variable	Scale (10-yr model)	Unit	Source dataset for anomaly calculation	Source dataset variable name	Years	Native spatial resolution
Heat Index Anomaly	N/A	°Celsius	ERA5-Land Hourly	Temperature_2m Dewpoint_temperture_2m	1970-2023	11,132 m^2
Air Temperature Anomaly	N/A	°Celsius	ERA5-Land Hourly	Temperature_2m	1970-2023	11,132 m^2
Relative Humidity Anomaly	N/A	Percentage	ERA5-Land Hourly	Temperature_2m Dewpoint_temperture_2m	1970-2023	11,132 m^2
Eastward Wind Anomaly	N/A	Meters per second	ERA5-Land Monthly Aggregated	U_component_of_wind_1 0m	1970-2023	11,132 m^2
Northward Wind Anomaly	N/A	Meters per second	ERA5-Land Monthly Aggregated	V_component_of_wind_1 0m	1970-2023	11,132 m^2
Latent Heat Flux Anomaly	$*10^{-6}$	Joules per meter squared	ERA5-Land Monthly Aggregated	Surface_latent_heat_flux_ sum	1970-2023	11,132 m^2
Absorbed Sunlight Anomaly	$*10^{-6}$	Joules per meter squared	ERA5-Land Monthly Aggregated	Surface_net_solar_radiati on_sum	1970-2023	11,132 m^2
Thermal Radiation Anomaly	$*10^{-6}$	Joules per meter squared	ERA5-Land Monthly Aggregated	Surface_net_thermal_radi ation_sum	1970-2023	11,132 m^2
Sensible Heat Flux Anomaly	$*10^{-6}$	Joules per meter squared	ERA5-Land Monthly Aggregated	Surface_sensible_radiatio n_sum	1970-2023	11,132 m^2
Sunlight Anomaly	$*10^{-6}$	Joules per meter squared	ERA5-Land Monthly Aggregated	Surface_solar_radiation_d ownwards_sum	1970-2023	11,132 m^2
Evaporation Anomaly	*10	Meters of equivalent water	ERA5-Land Monthly Aggregated	Total_evaporation_sum	1970-2023	11,132 m^2
Surface Pressure Anomaly	$*10^{-1}$	Pascals	ERA5-Land Monthly Aggregated	Surface_pressure	1970-2023	11,132 m^2
Precipitation Anomaly	*10	Meters	ERA5-Land Monthly Aggregated	Total_precipitation_sum	1970-2023	11,132 m^2
Transpiration Anomaly	$*10^{3}$	Meters of equivalent water	ERA5-Land Monthly Aggregated	Evaporation_from_vegeta tion_transpiration_sum	1970-2023	11,132 m^2

Table S12. Climate variables used in the analysis.

Supplemental References

Bartoń, K. (2024, June 22). MuMIn: Multi-Model Inference (Version 1.48.4). Retrieved from https://cran.r-project.org/web/packages/MuMIn/index.html

Bates, D., Mächler, M., Bolker, B., & Walker, S. (2015). Fitting Linear Mixed-Effects Models Using lme4. *Journal of Statistical Software*, *67*, 1–48. https://doi.org/10.18637/jss.v067.i01

Bates, D., Maechler, M., Bolker, B., Walker, S., Christensen, R. H. B., Singmann, H., et al. (2024, July 3). lme4: Linear Mixed-Effects Models using “Eigen” and S4 (Version 1.1-35.5). Retrieved from https://cran.r-project.org/web/packages/lme4/index.html

Daly, C., Smith, J. I., & Olson, K. V. (2015). Mapping Atmospheric Moisture Climatologies across the Conterminous United States. *PLOS ONE*, *10*(10), e0141140. https://doi.org/10.1371/journal.pone.0141140

Desai, M., Navale, A., & Dhorde, A. G. (2021). Evolution of Heat Index (HI) and Physiological Equivalent Temperature (PET) Index at Mumbai and Pune Cities, India. *MAUSAM*, *72*(4), 915–934. https://doi.org/10.54302/mausam.v72i4.3558

Fox, J., Weisberg, S., Price, B., Adler, D., Bates, D., Baud-Bovy, G., et al. (2023, March 30). car: Companion to Applied Regression (Version 3.1-2). Retrieved from https://cran.r-project.org/web/packages/car/index.html

Gorelick, N., Hancher, M., Dixon, M., Ilyushchenko, S., Thau, D., & Moore, R. (2017). Google Earth Engine: Planetary-scale geospatial analysis for everyone. *Remote Sensing of Environment*, *202*, 18–27. https://doi.org/10.1016/j.rse.2017.06.031

Greenlund, K. J., Lu, H., Wang, Y., Matthews, K. A., LeClercq, J. M., Lee, B., & Carlson, S. A. (2022). PLACES: Local Data for Better Health. *Preventing Chronic Disease*, *19*, E31. https://doi.org/10.5888/pcd19.210459

Lüdecke, D., Ben-Shachar, M. S., Patil, I., Waggoner, P., & Makowski, D. (2021). performance: An R Package for Assessment, Comparison and Testing of Statistical Models. *Journal of Open Source Software*, *6*(60), 3139. https://doi.org/10.21105/joss.03139

Lüdecke, D., Makowski, D., Ben-Shachar, M. S., Patil, I., Waggoner, P., Wiernik, B. M., et al. (2024, July 18). performance: Assessment of Regression Models Performance (Version 0.10.8). Retrieved from https://cran.r-project.org/web/packages/performance/index.html

Muñoz Sabater, J. (2019). ERA5-Land monthly averaged data from 1950 to present. [Https://doi.org/10.24381/cds.68d2bb30]. Copernicus Climate Change Service (C3S) Climate Data Store (CDS). Retrieved from https://developers.google.com/earth-engine/datasets/catalog/ECMWF_ERA5_LAND_HOURLY#description

National Oceanic and Atmospheric Administration. (n.d.). State Climate Extremes Committee (SCEC) | Records | National Centers for Environmental Information (NCEI). Retrieved August 1, 2024, from https://www.ncei.noaa.gov/access/monitoring/scec/records

National Weather Service. (n.d.). Heat Index Equation. Retrieved March 14, 2024, from https://www.wpc.ncep.noaa.gov/html/heatindex_equation.shtml

Pierannunzi, C., Xu, F., Wallace, R. C., Garvin, W., Greenlund, K. J., Bartoli, W., et al. (2016). A Methodological Approach to Small Area Estimation for the Behavioral Risk Factor Surveillance System. *Preventing Chronic Disease*, *13*, E91. https://doi.org/10.5888/pcd13.150480

Pollard, T. J., Johnson, A. E. W., Raffa, J. D., & Mark, R. G. (2018). tableone: An open source Python package for producing summary statistics for research papers. *JAMIA Open*, *1*(1), 26–31. https://doi.org/10.1093/jamiaopen/ooy012

Rajib, M., Mortuza, R., Selmi, S., Ankur, A., & Rahman, Md. M. (2011). Increase of Heat Index over Bangladesh: Impact of Climate Change. *International Scholarly and Scientific Research & Innovation*, *2011*, 5.

Rothfusz, L. P. (1990). *The Heat Index "Equation" (or, More Than You Ever Wanted to Know About Heat Index)* (Technical Attachment No. SR 90-23). National Weather Service. Retrieved from https://www.weather.gov/media/ffc/ta_htindx.PDF

Rural-Urban Commuting Area Codes | Economic Research Service. (n.d.). Retrieved October 30, 2025, from https://www.ers.usda.gov/data-products/rural-urban-commuting-area-codes

Steadman, R. G. (1979). The Assessment of Sultriness. Part I: A Temperature-Humidity Index Based on Human Physiology and Clothing Science. *Journal of Applied Meteorology and Climatology*, *18*(7), 861–873. https://doi.org/10.1175/1520-0450(1979)018<0861:TAOSPI>2.0.CO;2

Wang, Y., Holt, J. B., Zhang, X., Lu, H., Shah, S. N., Dooley, D. P., et al. (2017). Comparison of Methods for Estimating Prevalence of Chronic Diseases and Health Behaviors for Small Geographic Areas: Boston Validation Study, 2013. *Preventing Chronic Disease*, *14*, E99. https://doi.org/10.5888/pcd14.170281

Wei, T., & Simko, V. (2021). An Introduction to corrplot Package (Version 1.47.5). Retrieved from https://cran.r-project.org/web/packages/corrplot/vignettes/corrplot-intro.html

Wickham, H., Averick, M., Bryan, J., Chang, W., McGowan, L., François, R., et al. (2019). Welcome to the Tidyverse. *Journal of Open Source Software*, *4*(43), 1686. https://doi.org/10.21105/joss.01686

Wickham, H., Chang, W., Henry, L., Pedersen, T., Takahashi, K., Wilke, C., et al. (2023). Create Elegant Data Visualisations Using the Grammar of Graphics (Version 3.4.4). Retrieved from https://ggplot2.tidyverse.org/

Williams, E., Funk, C., Peterson, P., & Tuholske, C. (2024). High resolution climate change observations and projections for the evaluation of heat-related extremes. *Scientific Data*, *11*(1), 261. https://doi.org/10.1038/s41597-024-03074-w

Yoshida, K., Bartel, A., Chipman, J. J., Bohn, J., McGowan, L. Da., Barrett, M., & Christensen, R. H. B. (2022, April 15). tableone: Create "Table 1" to Describe Baseline Characteristics with or without

Propensity Score Weights (Version 0.13.2). Retrieved from https://cran.r-project.org/web/packages/tableone/index.html

Zhang, X., Holt, J. B., Yun, S., Lu, H., Greenlund, K. J., & Croft, J. B. (2015). Validation of Multilevel Regression and Poststratification Methodology for Small Area Estimation of Health Indicators From the Behavioral Risk Factor Surveillance System. *American Journal of Epidemiology*, *182*(2), 127–137. https://doi.org/10.1093/aje/kwv002
